# Lactate supports Treg function and immune balance via MGAT1 effects on N-glycosylation in the mitochondria

**DOI:** 10.1172/JCI175897

**Published:** 2024-09-12

**Authors:** Jinren Zhou, Jian Gu, Qufei Qian, Yigang Zhang, Tianning Huang, Xiangyu Li, Zhuoqun Liu, Qing Shao, Yuan Liang, Lei Qiao, Xiaozhang Xu, Qiuyang Chen, Zibo Xu, Yu Li, Ji Gao, Yufeng Pan, Yiming Wang, Roderick O’Connor, Keli L. Hippen, Ling Lu, Bruce R. Blazar

**Affiliations:** 1 Jiangsu Key Laboratory of Organ transplantation and transplant immunology; Research Unit of Liver Transplantation and Transplant Immunology, Chinese Academy of Medical Sciences; Hepatobiliary Center, the Affiliated Hospital of Xuzhou Medical University, Xuzhou, China.; 2 Collaborative Innovation Center for Cancer Personalized Medicine, Nanjing Medical University, Nanjing, China.; 3 Center for Cellular Immunotherapies and Department of Pathology and Laboratory Medicine, Perelman School of Medicine of the University of Pennsylvania, Philadelphia, Pennsylvania, USA.; 4 University of Minnesota, Department of Pediatrics, Division of Blood & Marrow Transplant & Cellular Therapy, Minneapolis, Minnesota, USA.

**Keywords:** Immunology, Metabolism, T cells

## Abstract

Current research reports that lactate affects Treg metabolism, although the precise mechanism has only been partially elucidated. In this study, we presented evidence demonstrating that elevated lactate levels enhanced cell proliferation, suppressive capabilities, and oxidative phosphorylation (OXPHOS) in human Tregs. The expression levels of Monocarboxylate Transporters 1/2/4 (MCT1/2/4) regulate intracellular lactate concentration, thereby influencing the varying responses observed in naive Tregs and memory Tregs. Through mitochondrial isolation, sequencing, and analysis of human Tregs, we determined that α-1,3-Mannosyl-Glycoprotein 2-β-N-Acetylglucosaminyltransferase (MGAT1) served as the pivotal driver initiating downstream N-glycosylation events involving progranulin (GRN) and hypoxia-upregulated 1 (HYOU1), consequently enhancing Treg OXPHOS. The mechanism by which MGAT1 was upregulated in mitochondria depended on elevated intracellular lactate that promoted the activation of XBP1s. This, in turn, supported MGAT1 transcription as well as the interaction of lactate with the translocase of the mitochondrial outer membrane 70 (TOM70) import receptor, facilitating MGAT1 translocation into mitochondria. Pretreatment of Tregs with lactate reduced mortality in a xenogeneic graft-versus-host disease (GvHD) model. Together, these findings underscored the active regulatory role of lactate in human Treg metabolism through the upregulation of MGAT1 transcription and its facilitated translocation into the mitochondria.

## Abstract 2

Lactate promotes the OXPHOS of human Tregs by inducing N-glycosylation in mitochondria via MGAT1, whose transcription by XBP1s and translocation by TOM70.

## Graphical abstract

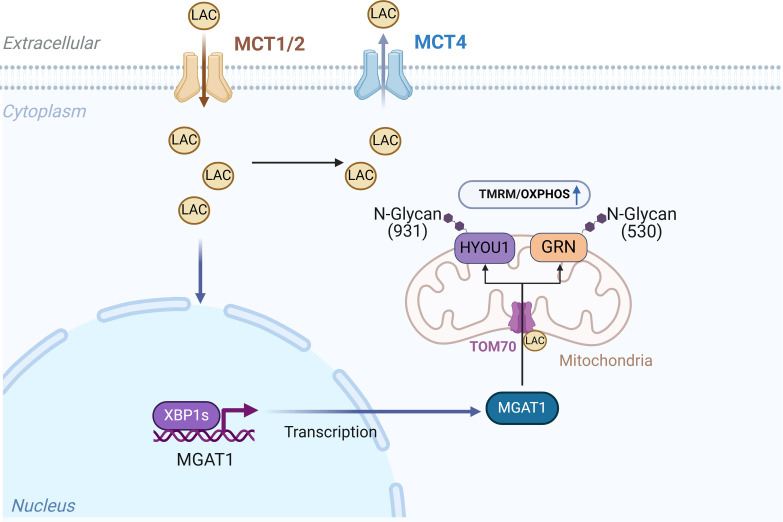

## Introduction

Tregs modulate the immune system, maintain balance and tolerance to antigens, and prevent autoimmune and alloimmune diseases, including graft-versus-host disease (GvHD) (1–3). The master transcription factor FOXP3 is essential for Treg differentiation and suppressor function (4–8). Tregs play a vital role in limiting immune-mediated inflammation by inhibiting effector T cell proliferation and generating cytokines through multiple mechanisms (9). Treg suppression mechanisms remain incompletely defined but include the production and release of immune suppressive metabolic catabolites and cytokines, IL-2 consumption, inhibitory receptor upregulation, B7 ligand transendocytosis, and cytolysis of antigen-presenting cells (10, 11).

There is an indisputable link between cellular metabolism and functional output in the immune system. Abnormal participation in metabolic pathways of immune cell subsets can result in problems such as abnormal function, loss of homeostasis, and disease progression. Procaccini et al. reported that glycolysis and fatty acid oxidation (FAO) are essential conditions for the expansion of human Treg cells in vitro (12). Cellular metabolism is critical for Treg epigenetic modification. De Rosa et al. reported that glycolysis controls induced Treg suppressive function via Foxp3-exon 2 (13).

Increased lactate production in the tumor microenvironment favors Treg survival, contributing to a high tumor Treg–to–T effector cell ratio and suppressor function (11). We have previously shown that lactate increases the differentiation of Tregs through lactylation on membrane-organizing extension spike protein MOESIN and enhances Treg TGF-β signaling (14). Kumagai et al. reported that lactate promotes Treg function by enhancing NFAT1 translocation and PD-1 transcription (15). Concerning metabolic effects, previous studies revealed that lactate acts as a substrate that participates in the tricarboxylic acid cycle (16). Here, we show that lactate also regulates mitochondrial function through controlling molecular pathways such as regulating transcription and translocation of MGAT1. It is reported that MGAT1 facilitates N-glycosylation of glucose transporter 1 (GLUT1) and increases the level of GLUT1 protein (17). Our study found that MGAT1 expression increased substantially in both naive Treg (TregN) and memory Treg (TregM) after lactate treatment and that the transcriptional factor XBP1s upregulated MGAT1 expression. More precisely, defining how lactate controls Treg metabolism could be of crucial therapeutic benefit for restoring autoimmune homeostasis, augmenting antitumor immunotherapy, and aiding in the development of new approaches to immunosuppression.

## Results

### Lactate promotes suppressive function and oxidative phosphorylation of TregN and TregM.

To explore the effect of high-concentration lactate on TregN (CD4^+^25^+^127^–^45RA^+^) and TregM (CD4^+^25^+^127^–^38^–^45RA^–^) ex vivo expansion, TregN and TregM from human PBMCs were initially sorted, expanded, and banked as previously published (Supplemental Figure 1A; supplemental material available online with this article; https://doi.org/10.1172/JCI175897DS1) (2). Both TregN and TregM presented compatible purity, expansion ability, and Treg-specific demethylated region (TSDR) level during the stimulation process (Table 1).

**Figure 1:**
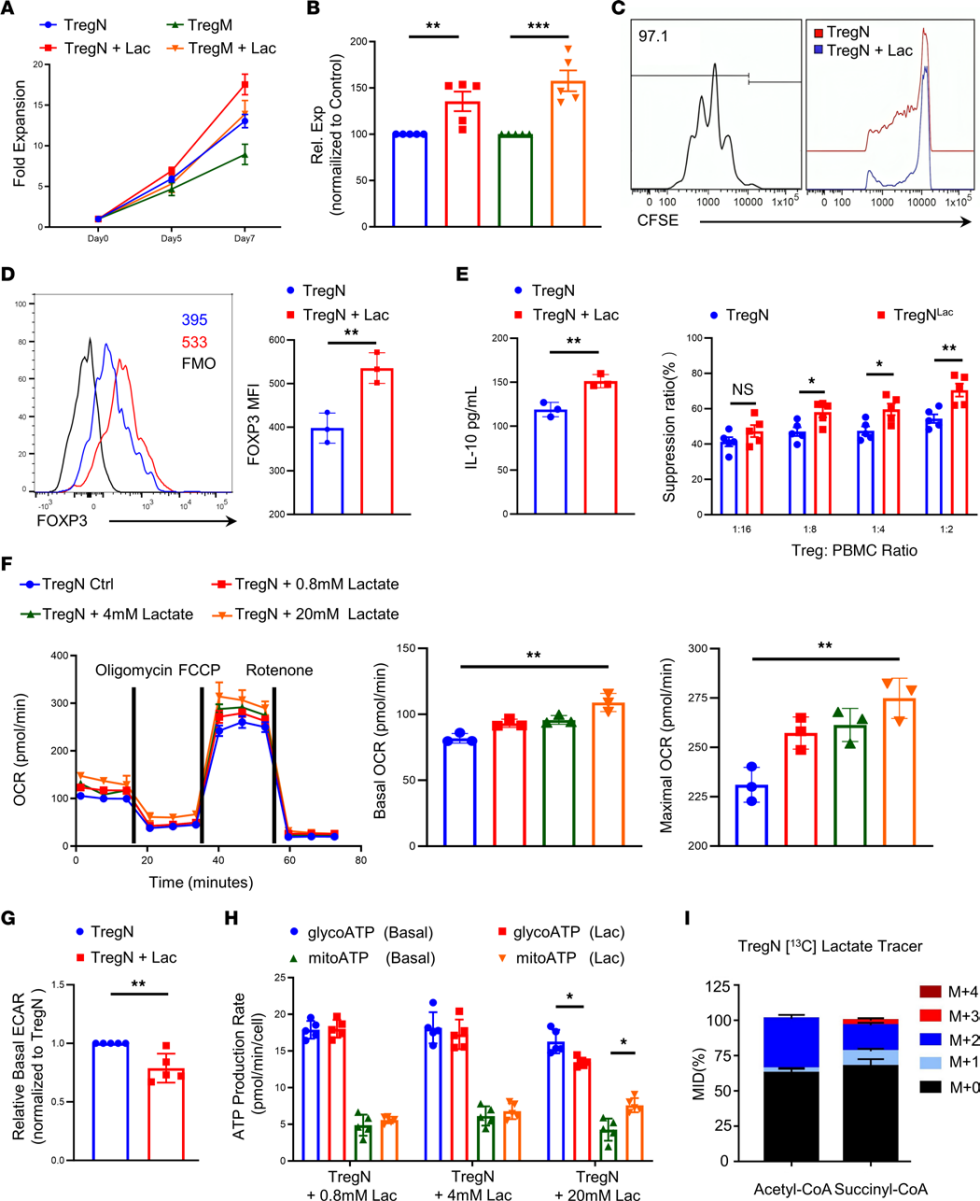
Lactate promotes suppressive function and OXPHOS of TregN. Naive and memory Tregs were initially flow sort–purified from human PBMCs, expanded in high-dose IL-2 (300 U/mL) for 14 days, and then frozen in aliquots for future study. Tregs were then thawed and assayed as indicated. (**A**) Proliferation curve of restimulated TregN and TregM with and without 20 mM lactate treatment. (**B**) Expansion of TregN and TregM with and without 20 mM lactate treatment (the physiological range of normal tissue is 0.5–20 mM) on day 7. *n* = 5. (**C**) Inhibitory effect of Treg cells on PBMC proliferation by flow cytometry (TregN were stimulated with CD3/CD28 mAb-coated beads, and cocultured with CFSE labeled PBMC in X-VIVO 15 media with and without 20 mM lactate for 48 hours). *n* = 5. (**D**) MFI of FOXP3 in TregN with and without lactate treatment for 48 hours by flow cytometry. *n* = 3. (**E**) Secretion of IL-10 in TregN with and without lactate treatment for 48 hours by flow cytometry. *n* = 3. (**F**) OCR of TregN was measured by Seahorse assay (TregN were stimulated with CD3/CD28 mAb-coated beads and treated with different doses of lactate for 24 hours). (**G**) Basal extracellular acidification rate (ECAR) of TregN with and without lactate treatment for 24 hours. (**H**) Mitochondrial-derived and glycolysis-derived ATP production following different doses of lactate treatment in TregN by ATP rate assay. **F**–**H** are representative of more-than 6 donors. (**I**) Tricarboxylic acid derivative analysis showed TregN integrated lactate into the Krebs cycle (TregN were harvested after 1-day stimulation with CD3/CD28 mAb-coated beads and treated with Sodium L-lactate-^13^C for an additional 1 hour). Data are represented as mean ± SEM. 2-tailed Student’s *t* test (**B**–**E**, **G**, and **H**) or 1-way ANOVA (**F**). *P < 0.05, **P < 0.01, ***P < 0.001.

Banked TregN and TregM were thawed, restimulated, and expanded. The proliferative capacity was maintained under expansion conditions in a high lactate concentration (sodium lactate, 20 mM) for 7 days (Figure 1, A and B). To test Treg suppressor function in vitro, prelactate-treated Tregs were washed, rested, and cocultured with CFSE-labeled PBMCs. Analysis of TregN and TregM suppressor function indicated that lactate-treated TregN and TregM showed significantly higher suppressor function than control Tregs (Figure 1C for TregN and Supplemental Figure 2A for TregM). Additionally, the mean fluorescence intensity (MFI) of FOXP3 increased after lactate treatment for 48 hours (Figure 1D for TregN and Supplemental Figure 2B for TregM). Testing for the expression of several other Treg markers revealed that the expression of TIGIT and Ki67 was increased while the activation marker CD69 remained the same after lactate treatment for 48 hours (Supplemental Figure 3A for TregN and Supplemental Figure 3B for TregM). The secretion of inhibitory cytokines is an essential function of Tregs. We found the secretion of IL-10 increased after lactate treatment in both Treg subsets (Figure 1E for TregN and Supplemental Figure 2C for TregM).

To explore the metabolic effects of lactate, stimulated TregN and TregM were treated with different lactate concentrations overnight before a mitostress extracellular flux assay. Consistent with the results of suppression assays, lactate at 20 mM, but not lower concentrations (0.8 or 4 mM), increased basal and maximal oxygen consumption rates (OCRs) in both Treg subsets (Figure 1F for TregN and Supplemental Figure 2D for TregM). In contrast, lactate (20 mM) reduced basal ECAR of both Tregs (Figure 1G for TregN and Supplemental Figure 2E for TregM). The results of the ATP rate assay also showed increased mitochondrial-derived ATP production and decreased glycolysis-derived ATP production following lactate treatment (Figure 1H for TregN and Supplemental Figure 2F for TregM). To assess the fate of extracellular lactate on activated TregN and TregM, cells were stimulated overnight with CD3/28 beads, and ^13^C-lactate (10 mM) was added for the final hour. These experiments found that lactate was rapidly incorporated into acetyl-CoA and succinyl-CoA (M+n represented metabolic intermediates with n ^13^C-labeled carbons), direct evidence that lactate could enter the tricarboxylic acid (TCA) cycle to increase oxidative phosphorylation (OXPHOS) (Figure 1I for TregN and Supplemental Figure 2G for TregM). Increased OXPHOS and reduced glycolysis were associated with augmented proliferation suppressor function of human TregN and TregM.

### A high lactate environment increases MGAT1 expression in TregN by mitochondrial proteomics.

The monocarboxylate transporter (MCT) family, located on the cell and mitochondrial membranes, is involved in lactate uptake and efflux. MCT1 (gene: *SLC16A1*) and MCT2 (gene: *SLC16A7*) have Km values preferential for import, whereas the high Km of MCT4 (*SLC16A3*) is more suited for lactate export. We found that the expression of MCT4 was higher in TregM than in TregN (Supplemental Figure 4, A–C). Meanwhile, the intracellular C^14^-sodium lactate concentration in TregN was higher than TregM, which indicated C^14^-sodium lactate surpassed TregM after the addition of isotope for 48 hours, while the intracellular content almost raised to the same level after the knockdown of *MCT4* via small hairpin RNA–carried (shRNA-carried) lentivirus (Supplemental Figure 4D).

**Figure 2:**
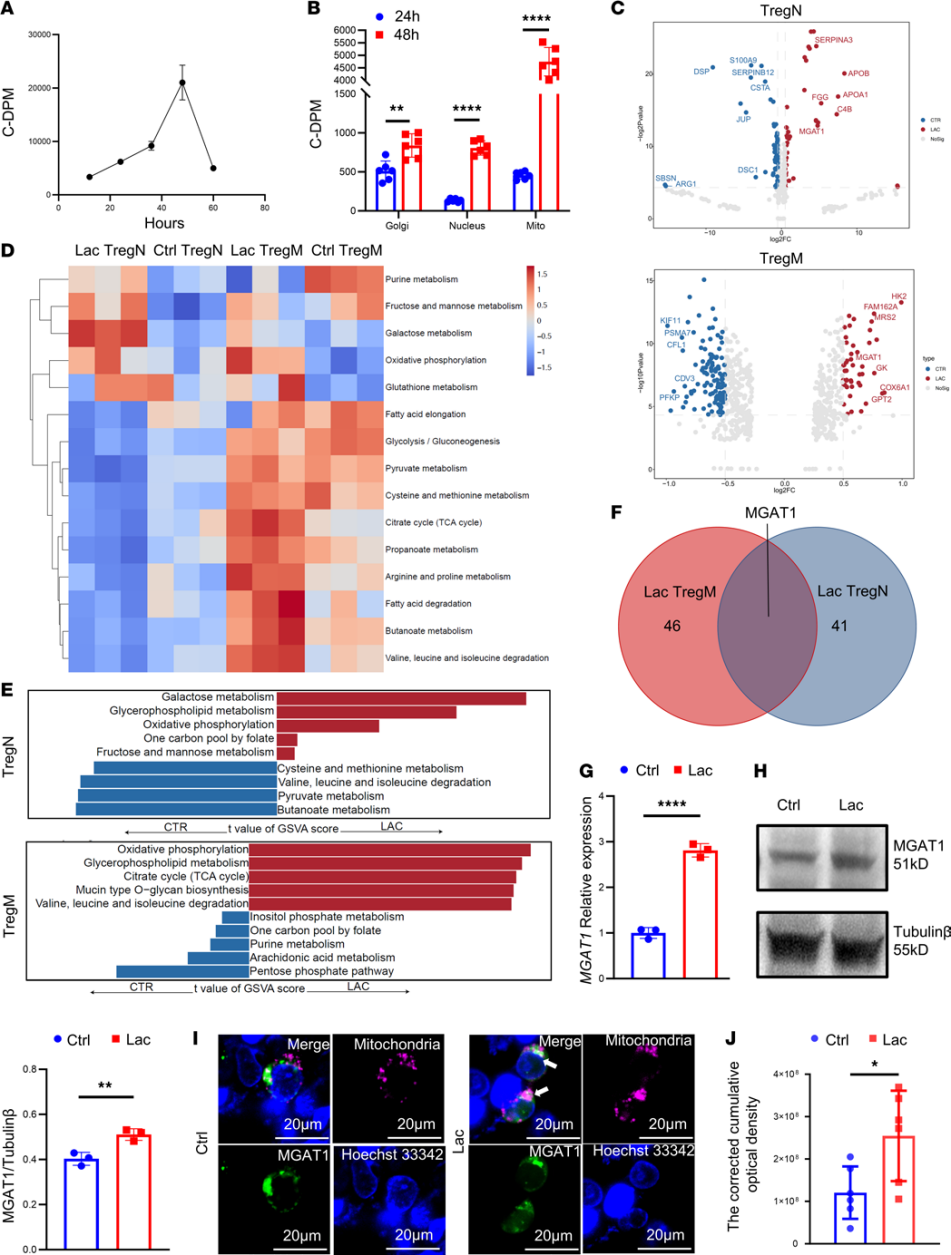
Mitochondrial proteomics show increased MGAT1 expression in TregN in the high lactate environment. (**A**) Isotope concentration peaked 48 hours after adding ^14^C-sodium lactate into the culture. *n* = 3. (**B**) Isotope concentration of ^14^C-sodium in each organelle increased significantly at 48 hours in TregN and was the highest in mitochondria. *n* = 6. (**C**) Differentially expressed proteins of TregN and TregM with 20 mM lactate treatment for 48 hours were performed by limma. (**D**) Strong TregN and TregM metabolic activation after lactate treatment through a heat map. *n* = 3. (**E**) Metabolic pathway scores were analyzed using limma to show the top 5 upregulated metabolic pathways after lactate treatment in TregN and TregM. (**F**) MGAT1 in TregN and TregM was upregulated after 20 mM lactate treatment for 48 hours from the intersection. (**G**) Expression of *MGAT1* mRNA was elevated in TregN after 20 mM lactate treatment for 48 hours. *n* = 3. (**H**) MGAT1 was elevated in TregN after 20 mM lactate treatment for 48 hours. *n* = 3. (**I**) Representative images of colocalization of MGAT1 and mitochondria were observed under laser scanning confocal microscopy in TregN (green and magenta merged white at the arrow). Scale bars: 20 μm.(**J**) The corrected cumulative optical density of colocalization in 2 groups. *n* = 6. The fluorescence intensity of each group was quantitatively analyzed for the brightest field of 3D video, and 6 copositioned fields were selected and additional blank areas were selected for background elimination. Then, the accumulated light intensity of the fluorescence images was analyzed using ImageJ (accumulated light density after correction is equal to the accumulated light density minus the area of cells to be measured multiplied by the average background light density). Data are represented as mean ± SEM. 2-tailed Student’s *t* test. *P < 0.05, **P < 0.01, ****P < 0.0001.

Since TregN showed a higher lactate concentration, which directly determined the more obvious cell response to lactate compared with TregM, we mainly chose TregN to explore mechanisms contributing to the observed lactate effects described above. Next, we investigated how lactate regulates TregN concentration at the intracellular level. ^14^C-sodium lactate was added and the isotope tracer study was performed to track the pathways lactate entered in TregN. Isotope concentration peaked at 48 hours after adding ^14^C-sodium lactate into the culture (Figure 2A). Therefore, we chose 24 hours and 48 hours to observe isotope concentration in the nucleus, Golgi, and mitochondria. Similar to the trend of total isotope changes in cells, the isotope concentration in each organelle increased significantly at 48 hours in TregN and was the highest in mitochondria (Figure 2B).

We further performed proteomics on purified mitochondria to examine changes after lactate treatment. The morphology of isolated mitochondria was depicted in Supplemental Figure 5A. The extracted mitochondrial protein quality was tested, and Western blot showed that the protein bands were uniform, distinguishable (Supplemental Figure 5B), and without other organelle contamination such as the Golgi apparatus and endoplasmic reticulum (Supplemental Figure 5C; COX IV as mitochondrial loading control). Differential expression analysis was performed by limma, and the differentially expressed proteins of TregN and TregM with 20 mM lactate treatment for 48 hours following stimulation using anti-CD3/CD28 mAb coated beads were obtained (Figure 2C). A total of 46 proteins increased substantially after lactate treatment for 48 hours in TregN and 41 in TregM. In addition, the ssGSEA algorithm was used to evaluate the activation status of metabolic pathways related to TregN and TregM. Based on unsupervised clustering, we found that TregN and TregM had evidence of metabolic activation after lactate treatment (Figure 2D). Metabolic pathway scores were analyzed using limma and showed that after lactate treatment, TregN specifically activated pathways associated with galactose metabolism, glycerophospholipid metabolism, OXPHOS, and other metabolic pathways. TregM also activated pathways leading to OXPHOS and glycerophospholipid metabolism, along with the TCA cycle, among others (Figure 2E). To find the most important proteins that regulate both TregN and TregM, we identified the intersection and found that MGAT1 was upregulated explicitly after treatment with lactate (Figure 2F). Western blot and qRT-PCR verified the elevated expression of MGAT1 in TregN (Figure 2, G and H) and TregM (Supplemental Figure 5, D and E).

To verify the promoting effects were specific to lactate rather than altered ionic concentration, we compared sodium lactate with sodium chloride (NaCl, 20 mM) in TregN. Consistent with Angelin et al., who reported that sodium chloride did not affect Treg phenotype and function (18), our results also showed that, as compared to the NaCl group, lactate promoted FOXP3 expression, MGAT1 expression, and OCR of TregN and that NaCl alone had no effect on either MGAT1 or OCR (Supplemental Figure 5, F–H).

Because most published papers indicated that MGAT1 is located in the Golgi apparatus and endoplasmic reticulum, we sought to further confirm the suborganelle localization of MGAT1 after lactate treatment. For that purpose, we constructed pEGFP-N1-MGAT1 and pCAG-mitochondrial-COX8-mKate2 (COX8 is a component of the cytochrome c oxidase) fusion plasmids and cotransfected 293T cells, our preffered tool cells. Laser scanning confocal microscopy showed that MGAT1 and mitochondrial COX8 protein colocalized in the mitochondria (green and magenta merged white) and lactate promoted the colocalization (Figure 2I). Additionally, 3D images (Supplemental Video 1 for the control group and Supplemental Video 2 for the lactate-treated group) were acquired to demonstrate that MGAT1 and mitochondria are not merely peripheral to one another. Quantification of colocalization was also carried out (Figure 2J).

### Lactate increases N-glycosylation of GRN and HYOU1 to promote TregN OXPHOS via MGAT1.

**Figure 3:**
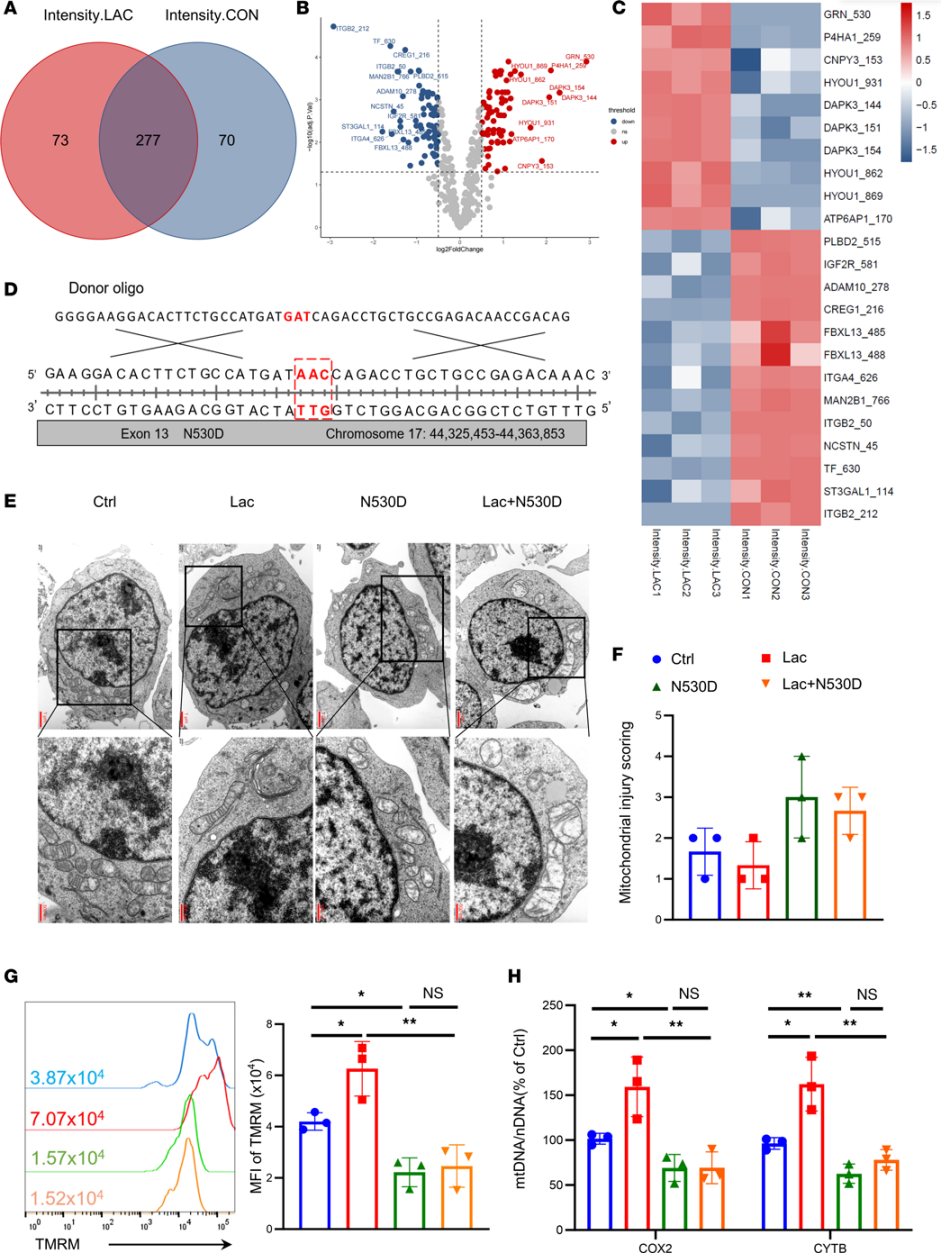
Lactate promotes Treg metabolism by GRN N-glycosylation. (**A**) TregN was cultured in a regular and 20 mM lactate-treated environment for 48 hours, and the mitochondria were isolated to perform mitochondrial N-glycosylation mass spectrometry. Differential N-glycosylation peptides were shown by limma via Venn diagram. (**B**) Specific differential N-glycosylation peptides in regular and lactate-treated groups by limma via Volcano map. (**C**) Total 9 upregulated and 13 down-regulated N-glycosylation peptides in lactate treated group by heat map. (**D**) CRISPR/Cas9 gene editing technology was used in Jurkat to mutate the Asparagine at GRN N530 to Aspartic acid in vitro. (**E**) Mitochondrial morphology under the transmission electron microscope in 4 groups after 48 hours of culture: Jurkat, lactate-treated Jurkat, GRN N530D mutation Jurkat, and lactate with GRN N530D mutation Jurkat. Obvious mitochondria swelling and disruption of mitochondrial crests were observed under the transmission electron microscope in the mutation and lactate-treated mutation group. Scale bars: 1 μm and 500nm. (**F**) Mitochondrial injury scoring of 4 groups in (**E**). *n* = 3. (**G**) TMRM fluorescence signal intensity of Jurkat in (**E**). *n* = 3. (**H**) Mitochondrial to nuclear DNA (mtDNA: nDNA) ratio of 4 groups in (**D**). *n* = 3. Data are represented as mean ± SEM. 1-way ANOVA (**G** and **H**). *P < 0.05, **P < 0.01.

MGAT1 can initiate complex N-linked carbohydrate formation and convert high-mannose to hybrid and complex N-glycans. Therefore, we performed mitochondrial N-glycosylation mass spectrometry. Compared with the control group, the N-glycosylation level of 73 peptides increased (Figure 3A). Among the increased peptides, 9 showed significant differences in N-glycosylation level (Lac/Ctrl > 1 and *P* < 0.05), with protein GRN showing the most considerable difference and HYOU1 showing the most increased peptides (Figure 3, B and C). Therefore, we selected GRN (N530) and HYOU1 (N931, ranked first among 3 N-glycosylation sites) for further research (Supplemental Figure 6, A and B). We first tested GRN and HYOU1 expression in the control and lactate groups and found that lactate did not change the protein expression (Supplemental Figure 6, C and D). The structure before and after N-glycosylation modification then was predicted using 3-dimensional modeling for GRN (N530) and HYOU1 (N931) (Supplemental Figure 6E for GRN and Supplemental Figure 6F for HYOU1).

GRN, a secreted glycoprotein, is abundantly expressed in various tissues and cell types with pleiotropic functions, including embryogenesis, inflammation, wound repair, neurodegeneration, and lysosome function. Recently, it was reported that GRN plays a vital role in maintaining mitochondrial homeostasis through controlling mitochondrial biogenesis and mitophagy in podocytes via PGRN-Sirt1-PGC-1α/FoxO1 signaling (19). To further examine the effect of GRN N530 N-glycosylation on Treg metabolism, we used CRISPR/Cas9 gene editing in Jurkat cells to mutate the Asparagine (N) at GRN N530 to Aspartic acid (D) in vitro (Figure 3D). First, we focused on changes in mitochondrial morphology. Unlike Jurkat alone and the lactate treated group, obvious mitochondrial swelling and disruption of mitochondrial crests was observed under the transmission electron microscope in the mutation and lactate-treated mutation group after culture for 48 hours (Figure 3E). Mitochondrial damage then was scored (Figure 3F) according to the rating scale (20) (Table 2). Tetramethylrhodamine Methyl Ester (TMRM) was used to measure mitochondrial membrane potential. Flow cytometry results showed that lactate promoted TMRM of Jurkat; however, the mutation decreased the intensity of TMRM, independent of the addition of lactate to mutated Jurkat (Figure 3G). Mitochondrial content was also assessed by mitochondrial DNA copy. Lactate increased the ratio of mitochondrial-to-nuclear DNA (mtDNA: nDNA) in Jurkat, which dropped sharply due to the mutation (Figure 3H).

Like GRN, CRISPR/Cas9 gene editing technology was also used in Jurkat T cells to mutate the Asparagine at HYOU1 N931 to Aspartic acid in vitro (Supplemental Figure 6G). HYOU1 is a stress-inducible chaperone molecule localized to the ER in numerous cell types (21). It is reported the localization and chaperoning functions of HYOU1 may not be limited to the ER but may also reside in the mitochondria, providing insight into the cytoprotective and antiapoptotic activities (22). Reactive oxygen species (ROS) are the product of oxidative stress. Flow cytometry showed that ROS intensity dropped with the addition of lactate in Jurkat and increased significantly after mutation (Supplemental Figure 6H). Additionally, mitochondrial membrane potential and mtDNA-to-nDNA ratio declined with HYOU1 N931 mutation (Supplemental Figure 6, I and J), suggesting a decreased mitochondrial function. To summarize, lactate promotes Treg metabolism by N-glycosylation of GRN and HYOU1 via MGAT1.

### Mitochondrial function of TregN declines after MGAT1 knockdown.

**Figure 4:**
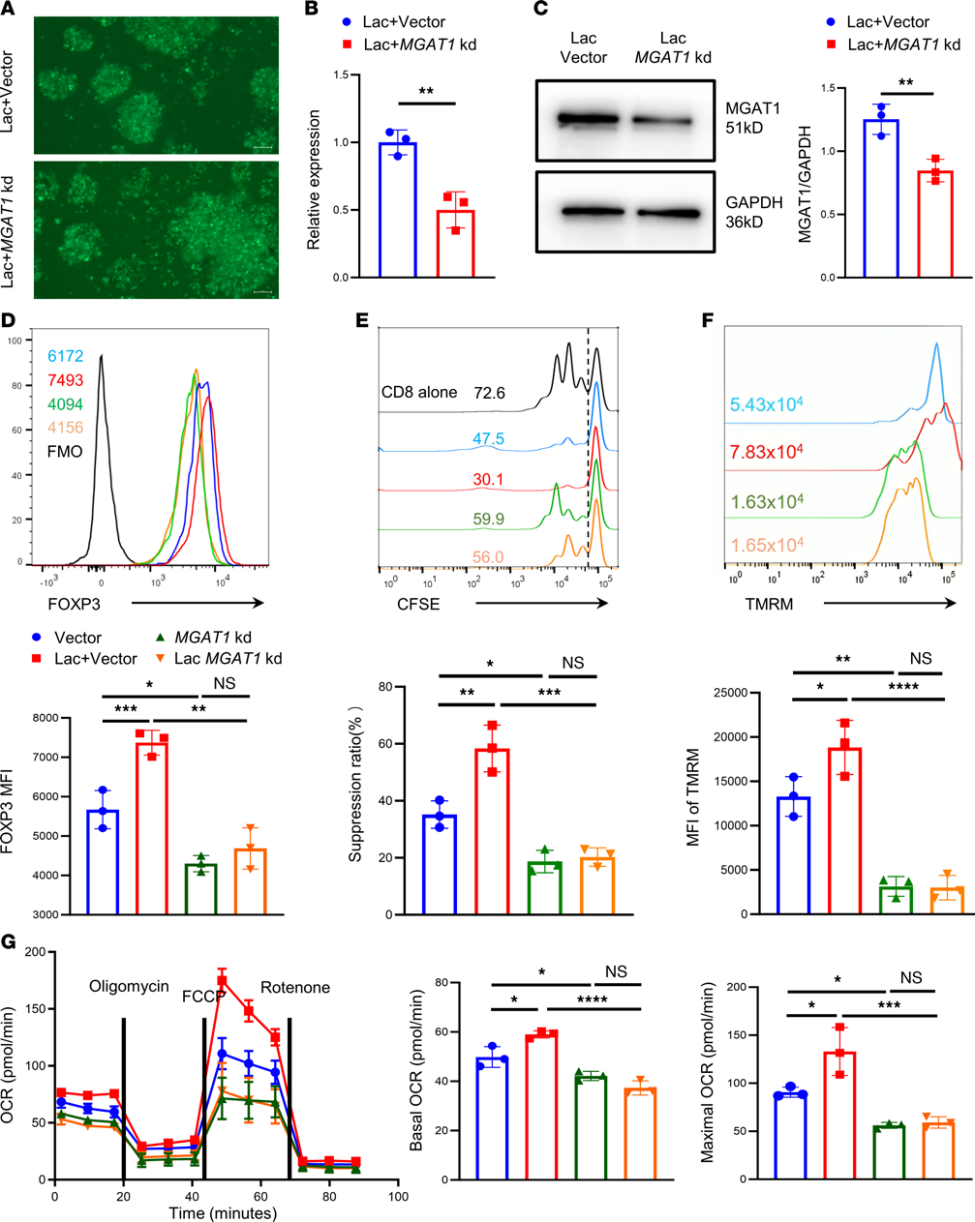
Mitochondrial function of TregN declines after *MGAT1* knockdown. (**A**) Transfection efficiency of lentivirus in lactate with lentiviral vector and lactate with *MGAT1* knockdown group for 3 days was shown by microscopy images in TregN. Scale bar: 100 μm. (**B**) Expression of *MGAT1* mRNA in groups in **A**. *n* = 3. (**C**) Expression of MGAT1 in groups in **A** by Western blot. *n* = 3. (**D**) Representative FOXP3 MFI by flow cytometry in 4 groups: lentiviral vector, lactate with lentiviral vector, *MGAT1* knockdown, and lactate with *MGAT1* knockdown group for 3 days. (**E**) Inhibitory function of TregN in 4 groups in **D** on CD8^+^ T cell proliferation (CD8: TregN = 2:1) by flow cytometry. *n* = 3. (**F**) Representative TMRM fluorescence signal intensity by flow cytometry in 4 groups in **D**. (**G**) OCR of TregM in 4 groups in **D** via cell metabolism measurement (Seahorse 1034 assay). Data are represented as mean ± SEM. 2-tailed Student’s *t* test (**B** and **C**) or 1-way ANOVA (**E**–**I**). *P < 0.05, **P < 0.01, ***P < 0.001, ****P < 0.0001.

To explore the role of MGAT1 in maintaining the mitochondrial function, oligonucleotides targeting MGAT1 were used for cloning shRNA-encoding sequences into the vector to construct lentivirus-based *MGAT1* knockdown plasmid. Microscopy images showed the transfection efficiency in lactate of the lentiviral vector and with *MGAT1* knockdown (Figure 4A for TregN and Supplemental Figure 7A for TregM). *MGAT1* mRNA and MGAT1 expression in mitochondria after *MGAT1* knockdown were tested (Figure 4, B and C for TregN and Supplemental Figure 7, B and C for TregM). Compared with the lactate-treated vector group, flow cytometry showed lower FOXP3 MFI and poor in vitro suppression function of Tregs in both *MGAT1* knockdown groups, whether or not lactate was added (Figure 4, D and E for TregN and Supplemental Figure 7, D–F for TregM). Regarding the mitochondrial function, lactate increased the intensity of TMRM in the vector group but significantly decreased the intensity after *MGAT1* knockdown (Figure 4F for TregN and Supplemental Figure 7, G and H for TregM). In addition, the Seahorse assay showed that *MGAT1* knockdown resulted in the decrease of basal and maximal OXPHOS levels in Tregs and counteracted lactate support of OXPHOS (Figure 4G for TregN and Supplemental Figure 7I for TregM).

### A high lactate environment elevates XBP1s, which facilitate MGAT1 transcription.

**Figure 5:**
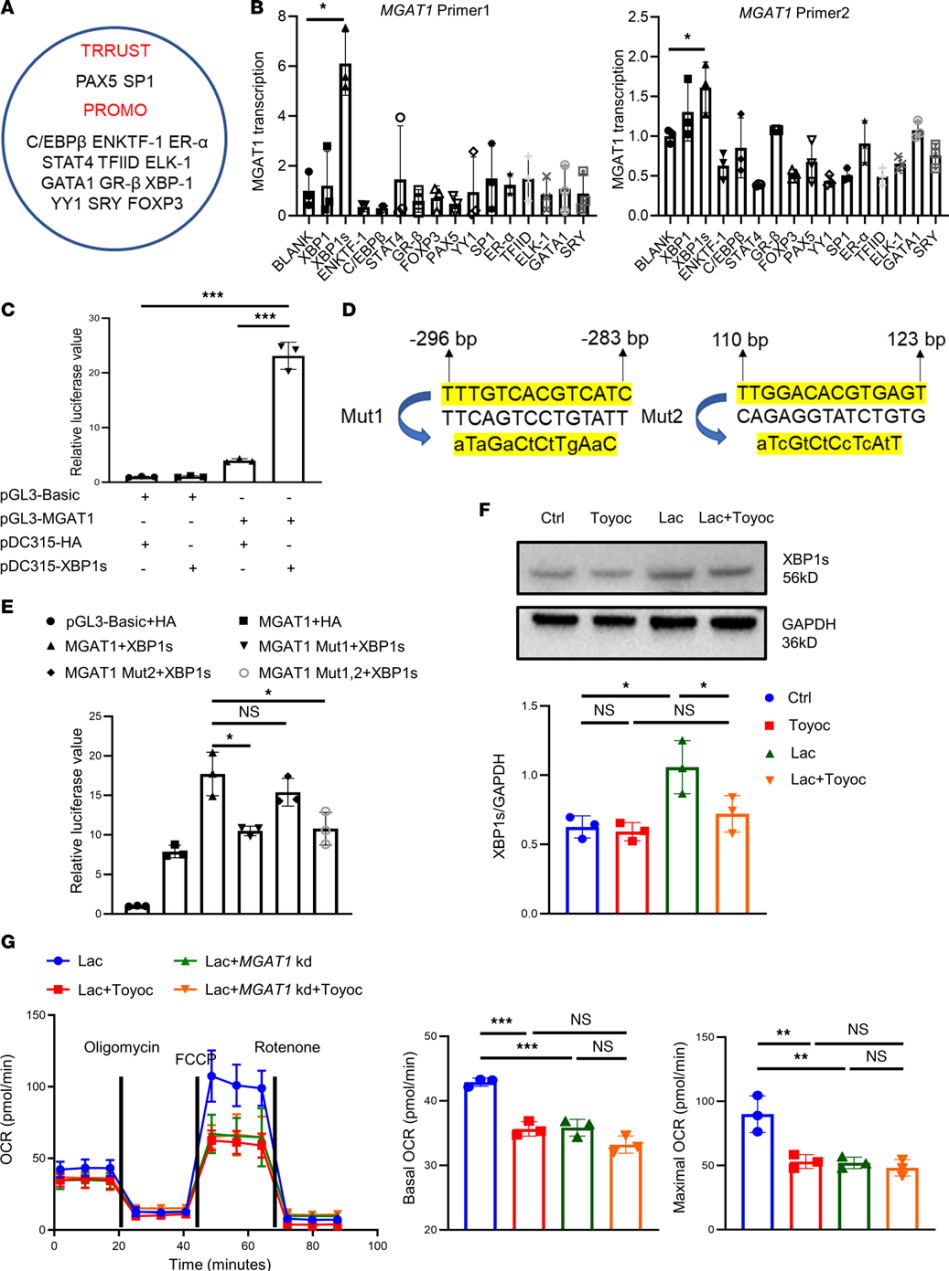
XBP1s is elevated to promote the transcription of MGAT1 in TregN in the high lactate environment. (**A**) Potential TFs of MGAT1 were found in TRRUST and PROMO databases. (**B**) Only XBP1s promoted the transcription of MGAT1 using 2 MGAT1 primers Among 15 TFs. *n* = 3. (**C**) Relative luciferase value after the addition of different combinations of pGL3-Basic, pGL3-MGAT1-promoter, Pdc315-HA, and Pdc315-XBP1s to HEK293 cells detected by microplate reader. *n* = 3. (**D**) 2 pGL3-MGAT1-promoter mutant constructs were designed according to 2 predicted binding sites from the JASPAR website. (**E**) Relative luciferase value after the addition of 2 pGL3-MGAT1-promoter Mutant constructs to HEK293 cells detected by a microplate reader. *n* = 3. (**F**) Expression of XBP1s in TregN in 4 groups for 48 hours by Western blot: control (ctrl), Toyocamycin, lactate, and Toyocamycin with lactate. *n* = 3. (**G**) OCR of TregN in 4 groups in 4 groups: Jurkat with lactate, Jurkat with lactate and Toyocamycin, *MGAT1* knockdown Jurkat with lactate, and *MGAT1* knockdown Jurkat with lactate and Toyocamycin. *n* = 3. Data are represented as mean ± SEM. 2-tailed Student’s *t* test (**B**, **C**, and **E**) or 1-way ANOVA (**F** and **G**). *P < 0.05, **P < 0.01, ***P < 0.001.

To better understand how *MGAT1* transcription increased after lactate treatment, we searched TRRUST and PROMO databases for potential transcription factors (TFs) that regulate *MGAT1*. A total of 15 TFs (XBP1s was included) were found; plasmids were constructed to determine whether any would promote *MGAT1* transcription in HEK293 cells (Figure 5A). Among 15 TFs, only XBP1s promoted the transcription of *MGAT1* using 2 MGAT1 primers (Figure 5B). The JASPAR website was used to search for the sequence of the *MGAT1* promoter region (from –2000 to 200 bp), which was then synthesized and cloned into a pGL3-basic luciferase reporter vector. Each MGAT1-luciferase reporter construct was delivered into HEK293 cells with an expression vector encoding XBP1s (pDC315-XBP1s) or its WT (pDC315-HA). In this system, pGL3-MGAT1-promoter alone induced only modest activation of MGAT1 expression. In contrast, a robust luciferase signal was detected when pGL3-MGAT1 promoter and pDC315-XBP1s were both present in the culture environment, indicating that XBP1s indeed significantly promoted the transcription of *MGAT1* (Figure 5C).

The JASPAR website predicted 2 potential binding sites according to the binding score: 15.4 for the first site and 8.6 for the second site (a score higher than 8 is more likely to become the binding site). Two pGL3-MGAT1 promoter mutant constructs were designed (Figure 5D). The results showed that the relative luciferase value dropped after adding mutant construct 1 while remaining essentially the same after adding mutant construct 2. Combined with the relative luciferase value of simultaneous mutation, we speculated that binding site 1 (between –296 bp and –283 bp) was most likely the binding site of XBP1s (Figure 5E).

Toyocamycin, an adenosine analogue produced by actinobacteria, is a potent inhibitor of ER stress–induced XBP1 mRNA splicing. We added Toyocamycin (80 nM) to the culture environment of TregN to determine whether XBP1s and MGAT1 expression would be affected. Western blot results showed that XBP1s expression in TregN did not decline with Toyocamycin treatment alone, while lactate increased XBP1s expression and it returned to the baseline level after adding Toyocamycin (Figure 5F). A similar condition was observed in the MGAT1 expression by laser scanning confocal microscopy (Supplemental Figure 8). Toyocamycin not only affects XBP1s, but also inhibits CDK9, among other targets. To verify that the effects were mediated through the XBP1s/MGAT1 axis instead of other targets, we constructed *MGAT1* knockdown Jurkat cells. Four conditions were tested for effects on OCR: lactate, lactate with Toyocamycin, *MGAT1* knockdown Jurkat cells with lactate, and *MGAT1* knockdown Jurkat cells with lactate and Toyocamycin. The results showed that basal and maximal OCR declined after adding Toyocamycin while the effect of toyocamycin was reduced when tested in *MGAT1* knockdown Jurkat cells. (Figure 5G).

### TOM70 is essential for the mitochondrial translocation of MGAT1 in a high lactate environment.

**Figure 6:**
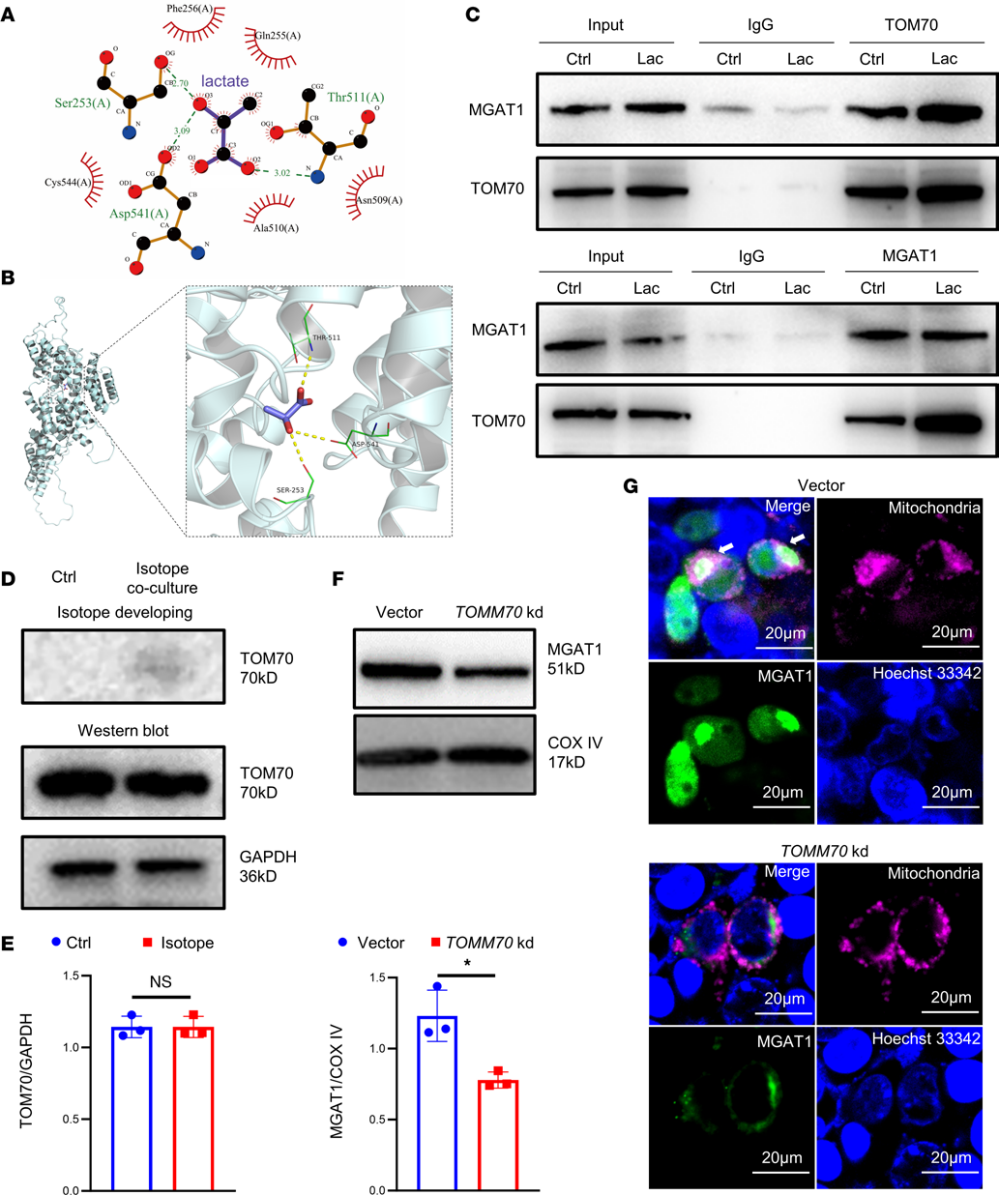
TOM70 is essential for mitochondrial translocation of MGAT1 in TregN in high lactate environment. (**A**) 2D interaction map of TOM70-lactate complex. (**B**) Based on the overall binding view and partial binding view of TOM70-lactate complexes were obtained based on docking, yellow dashed lines indicate hydrogen bonding, green dashed lines indicate amino acids forming hydrogen bonds with the lactate at the binding site, and purple stick shows lactate molecule. (**C**) Binding of MGAT1 and TOM70 before and after lactate treatment was tested by Co-IP. (**D**) Expression of TOM70 by Western blot and Autoradiography (^14^C-sodium lactate isotope) of TOM70 via isotype developing. (**E**) Analysis of Western blot in **D**. *n* = 3. (**F**) Expression of MGAT1 in mitochondria after *TOMM70* knockdown by Western blot. *n* = 3. (**G**) Colocalization of MGAT1 and mitochondria was observed under laser scanning confocal microscopy in TregN after *TOMM70* knockdown. Scale bars: 20 μm. Data are represented as mean ± SEM. 2-tailed Student’s *t* test. *P < 0.05.

Next, we explored the mechanism(s) responsible for the mitochondrial translocation of MGAT1. TOM70, encoded by *TOMM70*, is an import receptor of the outer mitochondrial membrane and part of the translocase of the outer membrane complex. To test the possibility that mitochondrial translocation of MGAT1 is associated with TOM70, we used AutoDock Vina (SMINA) software to conduct docking research on lactate, a small molecule, and TOM70 protein (negative values indicate the possibility of combination) (23). Compared with other TOM family proteins, the binding affinity of lactate and TOM70 protein was –4.4 kcal/mol, which meant that lactate and TOM70 protein had potential activity effects (Supplemental Figure 9A). In the complex of TOM70-lactate, lactate could bind to the pockets of TOM70 protein surrounded by amino acids, hydrogen bonding with SER253, ASP541, and THR511, while hydrophobic interaction was observed with PHE256, GLN255, ASN509, ALA510, and CYS544 (Figure 6, A and B and Supplemental Figure 9B). Co-IP verified that lactate promoted the binding of MGAT1 and TOM70 (Figure 6C). After adding ^14^C-sodium lactate into the culture of TregN for 48 hours, we used TOM70 antibody and corresponding secondary antibody to isolate TOM70 and found the isotope on the protein via autoradiography, revealing the binding of lactate to TOM70 (Figure 6, D and E). Oligonucleotides targeting *TOMM70* were used for cloning shRNA-encoding sequences into the vector to construct a lentivirus-based *TOMM70* knockdown plasmid that resulted in the decline of TOM70 expression after transfection (Supplemental Figure 9C). Western blot results showed that MGAT1 expression declined and laser scanning confocal microscopy showed the colocalization of MGAT1 and mitochondrial COX8 (a component of the cytochrome c oxidase) was reduced in the mitochondria after *TOMM70* knockdown (Figure 6, F and G). Further, basal and maximal OCR also declined after *TOMM70* knockdown (Supplemental Figure 9D).

### MGAT1 knockdown attenuates the therapeutic effect of Treg in a xenogeneic graft-versus-host disease model.

**Figure 7:**
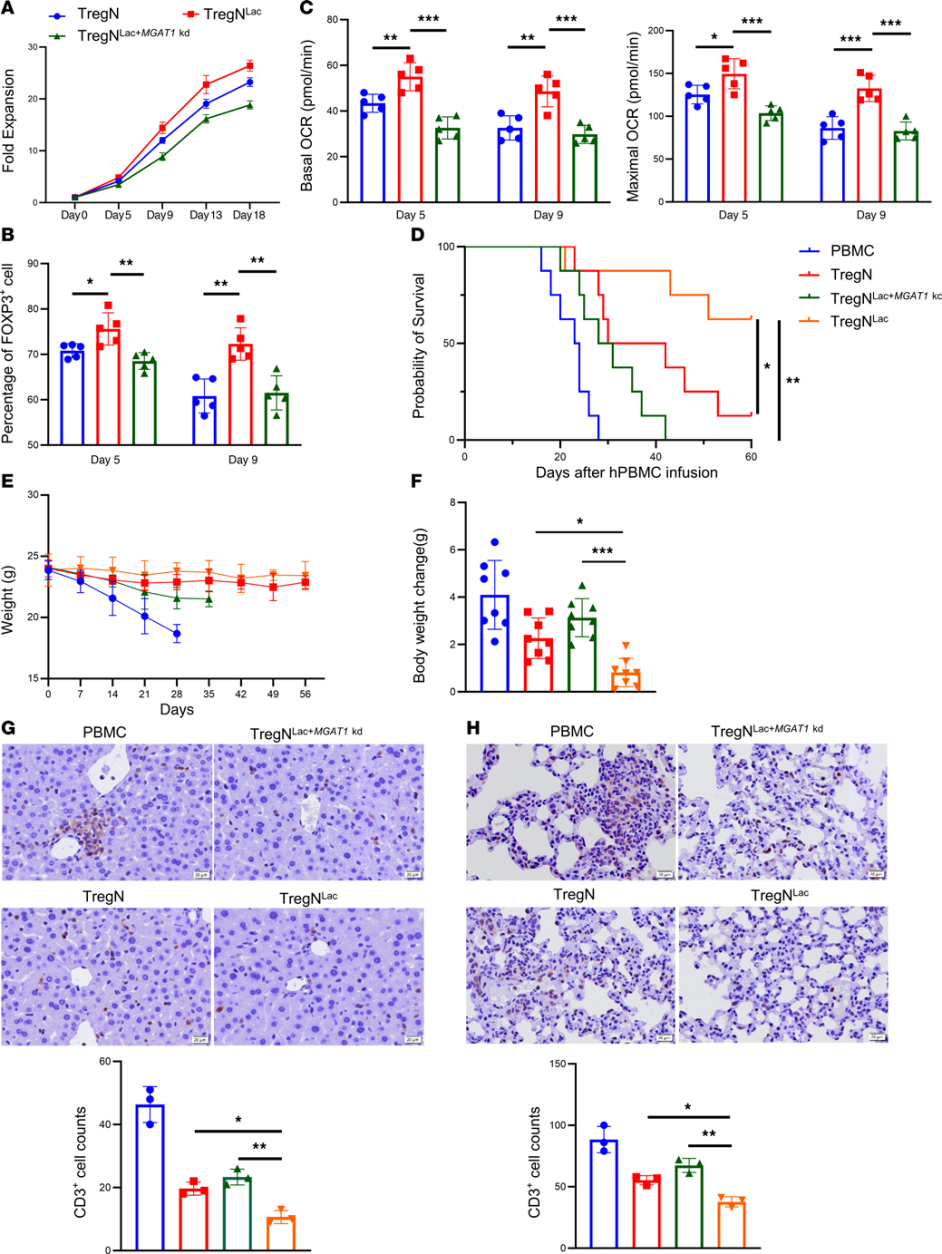
*MGAT1* knockdown attenuates the therapeutic effect of lactate-treated TregN on GvHD. (**A**) Cell proliferation of lactate-treated Tregs, normal Tregs, and *MGAT1* knockdown with lactate-treated Tregs on days 5, 9, 13, and 18. *n* = 3. (**B**) FOXP3 expression of Tregs in **A** on days 5, and 9. *n* = 5. (**C**) Basal and maximal OCR of Tregs in **A** on days 5 and 9. *n* = 5. (**D**) The survival of GvHD mice within 60 days injected with PBMC, PBMC with 1,087 untreated TregN, PBMC with lactate-treated TregN, and PBMC with lactate-treated *MGAT1* knockdown TregN via tail vein (10 million cells). *n* = 8. (**E**) Weight change of GvHD mice within 60 days in **D**. *n* = 8. (**F**) Representative histogram of Weight change of GvHD mice within 60 days in (**D**). *n* = 8. (**G**) CD3+ T cell infiltration in the liver of GvHD mice in (**D**) by immunohistochemistry. *n* = 3. (**H**) CD3+ T cell infiltration in the lung of GvHD mice in (**D**) by immunohistochemistry. Scale bars (**G** and **H**): 20 μm. *n* = 3. Data are represented as mean ± SEM. Statistical analysis was performed using 1-way ANOVA (**B**, **C**, **F**, **G**, and **H**) or 2-way ANOVA (**D**). *P < 0.05, **P < 0.01, ***P < 0.001.

Our in vitro findings that *MGAT1* knockdown Tregs were less functional than control Tregs prompted us to investigate whether *MGAT1* knockdown in Tregs mitigated the in vivo benefit of Tregs on host survival. Sorted Tregs were cocultured with anti-CD3/CD28 mAb beads and IL-2 for 9 days and restimulated with the same reagents for another 9 days (24). Compared with *MGAT1* knockdown, lactate treatment significantly increased the Treg proliferation rate (Figure 7A). Moreover, FOXP3 expression was higher in the lactate group on day 5 and day 9 while presenting more robust stability than the other 2 groups (Figure 7B). For in vivo applications, Tregs expanded long term need to be stable to exert their suppressive effect. Lactate-treated Tregs showed a higher and more stable OXPHOS level than control Tregs; this effect was abolished with *MGAT1* depletion (Figure 7C).

To assess the in vivo suppressive function of Tregs, xenogeneic GVHD studies were performed using Tregs expanded ex vivo for 13 days. Mice were randomly divided into 4 groups: PBMC, PBMC with untreated Treg, PBMC with lactate-treated Treg, and PBMC with lactate-treated, *MGAT1* knockdown Tregs. After 12-hour fasting, mice (*n* = 8 mice/group) received a single dose of 200 cGy of γ irradiation before the same-day injection of human PBMCs and Tregs (10 million cells per mouse). Unsurprisingly, lactate-treated Tregs significantly prolonged mouse survival and decreased weight loss compared with the untreated Treg group, while *MGAT1* knockdown in Tregs reversed this therapeutic effect (Figure 7, D–F for TregN and Supplemental Figure 10, A–C for TregM). Additionally, IHC was performed to evaluate the human cellular infiltration into GVHD target organs in vivo. We observed more CD3^+^ T lymphocyte infiltration in the liver and lung in the lactate-treated *MGAT1* knockdown Treg group compared with untreated and lactate-treated counterparts (Figure 7, G and H for TregN and Supplemental Figure 10, D and E for TregM). These data suggested that MGAT1 is essential for both Tregs to be therapeutic in a xenogeneic GvHD model induced by human PBMCs.

## Discussion

We found that lactate induced metabolic remodeling and improved the function of Tregs by promoting mitochondrial OXPHOS. Mitochondrial proteomics showed that after lactate treatment, only the MGAT1 expression was significantly increased in both TregN and TregM, and XBP1s upregulated this expression. We confirmed the colocalization of MGAT1 and mitochondria and found that MGAT1 entered mitochondria through molecular channel TOM70. During the process of culturing Tregs, we noted that the proliferation rate of TregN was significantly higher than that of TregM, suggesting a broader application prospect for TregN than TregM in Treg reinfusion therapy. Finally, we demonstrated the importance of lactate and MGAT1 in supporting in vivo Treg function in a xenogeneic GvHD model.

Our study was inspired by the immunosuppressive tumor microenvironment caused by high lactate (25, 26). The unique metabolic tumor microenvironment is manifested by glycolysis energy generation in an aerobic environment without utilizing OXPHOS and is associated with high lactate levels (Warburg effect). Angelin et al. found that Tregs could adapt to a high lactate environment by decreasing the use of Myc and glycolysis, increasing the level of OXPHOS, and promoting nicotinamide adenine dinucleotide (NAD^+^) oxidation (18). Lactate-induced increased NAD^+^ then impairs glycolysis and depletes glucose-derived serine by inhibiting the function of glyceraldehyde 3-phosphate dehydrogenase (GAPDH), thereby inhibiting T effector cells (Teff) proliferation and function (27). In addition, Comito et al. also found that lactate could suppress the tumor immune environment by inhibiting Th1 cells and increasing the number and function of Tregs (28). A recent study by Delgoffe and colleagues also showed that lactate metabolism is essential for Tregs in the tumor microenvironment, and blocking lactate uptake inhibits Treg proliferation and function in the tumor microenvironment, resulting in slower tumor growth and increased immunotherapy response (16). Thus, these studies suggested that high lactate could directly affect Treg metabolism to improve suppressor function.

In TregN and antigen-experienced TregM, we discovered that the metabolic enzyme MGAT1 is the pivotal driver initiating downstream N-glycosylation events involving GRN and HYOU1 that lead to enhanced OXPHOS. Glycosylation stands as one of the most familiar types of posttranslational modifications. The intricate array of glycosylation gives rise to a vast diversity of glycoproteins, glycan moieties, and glycolipids, subsequently imparting a dynamic and adaptable dimension to intra- and intercellular signaling regulation (29). Glycosylation is mainly divided into N-glycosylation and O-glycosylation. MGAT1 is related to the initiation of complex N-linked carbohydrate formation and high-mannose conversion to hybrid and complex N-glycans leading to our focus on N-glycosylation.

Nunes et al. reported that cells deficient in OXPHOS exhibited enhanced motility and migration characteristics, linked to altered glycosylation patterns and distinct membrane distribution of integrin-β1 (30). N-glycosylation and OXPHOS occur in different intracellular locations, with N-glycosylation primarily occurring in the ER and the Golgi apparatus, while OXPHOS occurs in mitochondria (31). Long et al. reported that changed N-glycosylation was critical for Treg function in vivo by assisting Treg migration to sites of immune priming rather than indispensable for intracorporal Treg inhibitory function (32). However, as a result of these distinct intracellular locations, little research has been reported on the relationship between N-glycosylation and OXPHOS. We believe that our study enriches knowledge of the known relationship between N-glycosylation and Tregs.

Among XBP1 functions, *XBP1* mRNA is processed by IRE1 during ER stress to its active form XBP1s, which can bind to the cis-regulatory X box located within the promoter sequences of histocompatibility complex class II genes, as well as the *MGAT1* promoter region. MGAT1 transcription is increased in a high lactate environment (33). The IRE1α-XBP1 pathway modulates T cell functionality in ovarian cancer through the regulation of mitochondrial dynamics and activity (34), demonstrating that XBP1s is also associated with mitochondrial function and tumor immunity. Still unknown is whether the observed XBP1s effects were related to ER stress, regulation of the unfolded protein response (35, 36), how IRE1 and other upstream molecules were altered, and the mechanisms by which XBP1s supported the terminal differentiation of B lymphocytes to Ig-producing plasma cells (37).

TOM70 is an import receptor of the outer mitochondrial membrane. The translocase of the outer membrane complex includes TOM70, which serves to recognize and facilitate the chaperone-dependent translocation of mitochondrial proteins from the cytosol into the mitochondria (38, 39). TOM70 also acts as a mitochondrial antiviral-signaling protein receptor and contributes to the relevant biological process by which the innate immune system fights viral infection (40). Mutations in TOM70 can result in deficiencies across multiple OXPHOS pathways, ultimately leading to severe conditions such as lactic acidosis, anemia, and developmental delays. (41). TOM70 also can regulate the immune system by engaging in the recruitment of PINK1 and the E3 ubiquitin ligase Parkin, playing a role in processes associated with Parkinson’s disease. Our studies found that TOM70 helped transport MGAT1 into mitochondria, adding another vital function to TOM70. In aggregate, these studies deepen our understanding of MGAT1 function and location in organelles.

Ex vivo addition of lactate and the discovery of MGAT1 may help enhance the efficacy of Treg reinfusion therapy. Because ex vivo Treg expansion efficiency is crucial in obtaining sufficient Treg numbers for adoptive therapy, investigators have sought to develop new methods to increase their proliferative capacity and improve their suppressive function. Our study found that high lactate ex vivo expansion conditions could enhance OXPHOS and immunosuppressive functions of Treg and, at the same time, inhibit the proliferative capacity and risk of Teff expansion that could limit the efficacy of Treg therapy. Enhancing the effectiveness of Treg infusion therapy may be simply achieved by adding lactate to the ex vivo culture media, which, in our case, resulted in superior effects of lactate-treated Treg on xenogeneic GvHD lethality.

## Methods

### Sex as a biological variable

Our study examined male and female animals, and similar findings are reported for both sexes.

### Human Treg isolation, expansion, and purification

PBMCs were isolated using Ficoll-Hypaque (Amersham Biosciences) from PB leukapheresis. PBMCs were placed in the autoMACS cell sorting system and the PosselDS program was run to sort CD25^+^ PBMCs and CD25^–^ PBMCs as described (2). CD25^+^ PBMCs were stained with CD4, CD25, CD127, CD38, and CD45RA and sorted via FACSAria (BD Biosciences) as naive Tregs (TregN; CD4^+^CD25^+^CD127^–^ CD45RA^+^) and memory Tregs (TregM; CD4^+^ CD25^+^ CD127^–^ CD38^–^ CD45RA^–^) (Supplemental Figure 1). For TregM isolation, CD38^+^ cells were excluded from the sort due to prior reports that this population could reprogram into Teff cells (42).TregN and TregM were stimulated and cultured in X-VIVO 15 (BioWhittaker) media with 10% human AB serum (Valley Biomedical). 300 IU/mL recombinant IL-2 (R&D Systems) was added every 2–3 days depending on the expansion of Tregs. Sort-purified Tregs were expanded 7–10 days after the primary stimulation, and 7–10 days for secondary stimulation. Anti-CD3/CD28 mAb-coated beads (Thermo Scientific; 11132D) were only added at the beginning of each stimulation. On day 10 ± 1, Treg aliquots were frozen for future use. When needed, Tregs were thawed and resuspended in X-VIVO 15 media at 0.25 M/mL with or without the reagent according to specific experimental design, stimulated right after thaw with anti-CD3/CD28 mAb-coated beads (at a 3:1 bead:cell ratio.) with cell density adjusted to 0.25 M/mL and incubated in 37°C.

### Cell Culture

Human embryonic kidney cell line 293T were purchased from Procell Life Science & Technology Company. Cells were cultured in DMEM (Gibco) supplemented with 10% FBS (Gibco) at 37°C with 5% CO_2_.

### RNA extraction and quantitative polymerase chain reaction

Total and mitochondrial RNA was extracted using Trizol (Invitrogen) following the manufacturer’s instructions and was reverse transcribed with a high-capacity cDNA reverse transcription kit (Roche). The mRNA levels were quantified by quantitative PCR with SYBR Green (Roche). Relative levels of *MGAT1* mRNA were quantified after normalization with corresponding levels of *GAPDH* mRNA. Primers used for human samples were as follows: *MGAT1* forward (F): CGCAAGTTCCAGGGCTACTAC; *MGAT1* reverse (R): CTTCAGCAGCGGATAGGTGG.

### Western blot

Cells were washed twice with PBS and dissected in radioimmunoprecipitation assay (RIPA) lysate buffer with protein phosphatase inhibitor and protease inhibitor, followed by protein concentration measurement using BCA. SDS-PAGE was used to separate proteins, then transferred to the PVDF membrane and then exposed. The following antibodies were used: recombinant Anti-SLC16A3/ MCT4 antibody (1:1,000, Abcam, ab308528), Tubulin (1:5,000, Bioworld, AP0064), recombinant Anti-Golgin 97 antibody (1:1,000, Abcam, ab308520), Anti-GRP94 antibody (1:2,000, Abcam, ab227293), Anti-COX IV antibody-Mitochondrial Loading Control (1μg/mL, Abcam, ab16056), recombinant Anti-MGAT1 antibody (1:2,000, Abcam, ab180578), Anti-GAPDH antibody[6C5]-Loading Control (5 μg/mL, Abcam, ab8245), Anti-Granulin antibody (1:1,000, Abcam, ab191211), Anti-ORP150 antibody (1:5,000, Abcam, ab134944), Anti-β-Actin antibody[mAbcam 8226]-Loading Control (1 μg/mL, Abcam, ab8226), recombinant Anti-XBP1 antibody (1:1,000, Abcam, ab220783), and recombinant Anti-TOM70 antibody (1:1,000, Abcam, ab289977).

### Flow cytometry and fluorescence-activated cell sorting

The stain followed standard flow stain protocol and FMO (Fluorescence Minus 1) was set for each fluorochrome. The acquisition was performed using a FACSCelesta (BD Biosciences) and data were analyzed using FlowJo 10.8.1 software. The following antibodies were used for staining: CD45-APC-Cy7 (BioLegend 304014), CD4-BUV395 (BioLegend, 344614), CD8-PE (BioLegend, 344705), CD25-BV605 (BioLegend, 356142), CD127-PE (BioLegend, 351304), FOXP3-PerCP5.5 (Biolegend, 320106), CD38-PE (BioLegend, 356614), Ki67-PECY7 (BioLegend, 350526), CD69-BV650 (BioLegend, 310934), and TIGIT (VSTM3)-BV711 (BioLegend, 372742).

### Suppression assay

PBMCs and CD8^+^ T cells were purified and labeled with the intracellular dye CFSE (Carboxyfluorescein diacetate succinimidyl ester) as described (43). Before the CFSE assay, Tregs or lactate-pretreated Tregs were washed with PBS and rested in a serum-free medium for 12 hours. Responders were stimulated with anti-CD3/CD28 mAb-coated beads with or without cultured Tregs (Treg/PBMCs or CD8 T cells at different ratios). On day 4, responders were labeled with antibodies to CD4 and CD8, and suppression was determined from Division Index using FlowJo software.

### Bioenergetic measurements

A Seahorse assay was used to measure the cell metabolism of Tregs by XF96 analyzer (Agilent Technologies). Tregs (2 × 10^5^ cells per well) were inoculated into CellTak (BD Biosciences) coated plates that were spun at 400*g*. To measure the extracellular acidification (ECAR), an indicator of glycolysis, 10 mM glucose, 2 μM oligomycin, and 50 mM 2-Deoxy-D-glucose (Agilent Glucostress kit) were sequentially added. The oxygen consumption rate (OCR), an indicator of the cellular respiration rate and mitochondrial function, was measured after the sequential addition of 1.5 μM oligomycin, 1.0 μM FCCP, 1 μM rotenone, and 1.8 μM antimycin A (Agilent Mitostress Kit). An ATP Rate Assay kit (Agilent Technologies) was used per the manufacturer’s guidebook.

### Mitochondria isolation

Isolation of mammalian mitochondria involved culturing cells on either a 100 mm or 150 mm plate. Mitochondria were extracted by homogenizing cells in 2 mL of mitochondrial isolation buffer (containing 210 mM mannitol, 70 mM sucrose, 10 mM HEPES, 1 mM EGTA, and 0.25% fatty acid-free BSA, pH adjusted to 7.2 with KOH) using a dounce homogenizer (320*g*, 20 strokes). Mitochondria were separated via differential centrifugation. Cell debris was sedimented at 800*g* for 10 minutes, and the supernatant was transferred to a fresh tube. This process was repeated until no cell debris remained. Subsequently, the supernatant was centrifuged at 11,000*g* for 15 minutes. The resulting mitochondrial pellet was then resuspended in 50–200 μL of BSA-free mitochondrial isolation buffer.

### Quantification of mitochondrial DNA content

Total cellular DNA was extracted using a FastPure Cell/Tissue DNA Isolation Mini Kit (Vazyme Biotech Co., Ltd.). DNA primers designed for cytochrome b (Cyt b) and cytochrome c oxidase subunit II (COII) were used to detect mitochondrial DNA (mtDNA) and β-actin to detect nuclear DNA (nDNA). Real-time PCR (RT-PCR) was performed using AceQ Universal SYBR Green qPCR Master Mix (Vazyme Biotech). Mitochondrial DNA and nuclear DNA were calculated by the 2^–ΔΔCT^ method, and the relative copy number of mitochondrial DNA was expressed as the ratio of mitochondrial to nuclear DNA (mtDNA:nDNA).

### Protein extraction and digestion

#### Protein extraction.

SDT buffer (details given below in Filter-aided protein preparation and peptide isolation) was added to the sample. The lysate was sonicated and then boiled for 15 minutes. After centrifuging at 14,000*g* for 40 minutes, the supernatant was quantified with the BCA Protein Assay Kit (Bio-Rad). The sample was stored at –80°C.

#### SDS-PAGE.

A total of 20 μg protein for each sample was mixed with 5× loading buffer and boiled for 5 minutes. Proteins were separated on 12.5% SDS-PAGE gel (constant current 14 mA, 90 minutes). Protein bands were visualized by Coomassie Blue R-250 staining.

#### Filter-aided protein preparation and peptide isolation.

Filter-aided protein preparation and peptide isolation was performed as per ref. 44. 200 μg of proteins for each sample were incorporated into 30 μL SDT buffer (4% SDS, 100 mM DTT, 150 mM Tris-HCl pH 8.0). The detergent, DTT, and other low-molecular-weight components were removed using UA buffer (8 M Urea, 150 mM Tris-HCl, pH 8.0) by repeated ultrafiltration (Microcon units, 10 kD). Then 100 μL iodoacetamide (100 mM IAA in UA buffer) was added to block reduced cysteine residues and the samples were incubated for 30 minutes in darkness. Filters were washed with 100 μL UA buffer 3 times and 100 μL 25 mM NH_4_HCO_3_ buffer twice. Finally, protein suspensions were digested with 4 μg trypsin (Promega) in 40 μL 25mM NH_4_HCO_3_ buffer overnight at 37°C, and the resulting peptides were collected as a filtrate. Peptides of each sample were desalted on C18 Cartridges (Empore SPE Cartridges C18 [standard density], bed I.D. 7 mm, volume 3 mL; Sigma-Aldrich), concentrated by vacuum centrifugation and reconstituted in 40 μL of 0.1% (v/v) formic acid. The peptide content was estimated by UV light spectral density at 280 nm using an extinction coefficient of 1.1 of 0.1% (g/l) solution calculated based on the frequency of tryptophan and tyrosine in vertebrate proteins.

### Lectin enrichment and deglycosylation in H2O18

The digested peptides were mixed with a lectin solution containing the combination of Concanavalin A (conA), wheat-germ agglutinin (WGA), and Ricinus communis agglutinin I (RCA 120) (Sigma-Aldrich), resulting in mixtures of peptides and lectins with a lectin-to-protein ratio of 1:2 (w/w). The mixtures were transferred to new YM-30 filter units (Sartorius). After 1 hour incubation at room temperature, the unbound peptides were eluted by centrifugation at 14,000*g* for 10 minutes. To avoid false positives caused by deamidation, captured peptides were washed with binding buffer and NH_4_HCO_3_ buffer in H_2_O^18^ (Cambridge Isotope Laboratories) 3 times before PNGaseF (Roche) was added. Finally, the filter units were transferred to a new tube, 3 μg PNGaseF in 40 μl 25 mM NH_4_HCO_3_ in H_2_O^18^ was added to the filter units, and the samples were incubated for 3 hours at 37°C. Deglycosylated peptides were eluted by centrifugation at 14,000g for 10 min after incubation.

### LC-MS/MS analysis

Liquid chromatography tandem mass spectrometry (LC-MS/MS) analysis was performed on a Q Exactive HF/HFX mass spectrometer (Thermo Fisher Scientific) coupled to Easy nLC (Thermo Fisher Scientific) for 120 minutes. The peptides were loaded onto a reverse phase trap column (Thermo Fisher Scientific Acclaim PepMap100, 100 μm*2 cm, nanoViper C18) connected to the C18-reversed phase analytical column (Thermo Fisher Scientific Easy Column, 10 cm long, 75 μm inner diameter, 3 μm resin) in buffer A (0.1% Formic acid) and separated with a linear gradient of buffer B (84% acetonitrile and 0.1% Formic acid) at a flow rate of 300 nL/min controlled by IntelliFlow technology (45). The mass spectrometer was operated in positive ion mode. MS data were acquired using a data-dependent top-10 method dynamically choosing the most abundant precursor ions from the survey scan (300–1800 m/z) for higher energy collisional-induced dissociation (HCD) fragmentation (45). The automatic gain control (AGC) target was set to 3 × 10^6^, and the maximum inject time to 10 milliseconds. Dynamic exclusion duration was 40.0 seconds. Survey scans were acquired at a resolution of 70,000 at m/z 200 and resolution for HCD spectra was set to 17,500 at m/z 200, and the isolation width was 2 m/z. The normalized collision energy was 30 eV, and the underfill ratio, which specifies the minimum percentage of the target value likely to be reached at maximum fill time, was defined as 0.1%. The instrument was run with peptide recognition mode enabled.

### Mitochondrial protein differential expression analysis by limma

The Limma package from R Bioconductor was used to complete quantile normalization of expression arrays and analyze differentially expressed mitochondrial proteins between 2 groups (*P* ≤ 0.05 and fold change ≥ 1.5).

### ssGSEA algorithm

Single-sample gene set enrichment analysis (ssGSEA) analysis, which calculates separate enrichment scores for each pairing of a sample and gene set was used to assess the gene score of every gene set for every sample.

### Point mutation

CRISPR/Cas9 technology was used to construct GRN (p.N530D) and HYOU1 (p.N931D) in Jurkat cell lines. Briefly, Cas9 protein, gRNA, and Donor vector were cotransfected into Jurkat cells, and then monoclonal cells were prepared by limiting dilution method. Finally, the DNA of different monoclonal cells was extracted and Sanger sequenced, and the monoclonal cell with the correct editing site sequence was selected.

### TRRUST, PROMO, and JASPAR databases

The MGAT1 promoter sequence was obtained through Gene using accession number 4245. We then entered the promoter sequence in 3 databases to search for possible transcription factors, followed by seeking 2 potential transcriptional binding sites on *MGAT1* promoter sequence in JASPAR database.

### Co-IP

Cells were washed twice, followed by being lysed with RIPA lysate, and then the protein concentration was determined via BCA Protein Assay Kit (Abcam). The lysates were clarified with agarose resin for antibody fixation, and the protein content in the filtrate was analyzed after elution of the complex. Western blot analysis was performed to determine the expression of binding proteins.

### Molecular docking

Protein 3D structure TOM70 used for docking was obtained by UniProt database (UniProt ID: O94826; ALPHAFOLD2 was used to predict conformation). 3D structures of small molecules of lactate were downloaded from the PubChem database (PubChem CID: 612) and its energy was minimized using AVOGADR 1.2.0 under the MMFF94 force field. SMINA software based on AutoDock Vina was used for molecular docking work. Before the docking, PyMol Academic Open Source version was used for hydrogenation of all receptor proteins. ADFRsuite 1.0 was then used to convert all the processed small molecules and receptor proteins into the PDBQT format required for SMINA docking. During docking, the PDBQT file of processed proteins and small molecules was used as the input file for docking using SMINA. The detail level of global search for docking was set to 32. The AUTOBOX parameter of SMINA was enabled to completely wrap the whole protein in the docking box, and the other parameters remained the default settings. Finally, the docking conformation with the highest output score was considered as the combination conformation, and PyMol Academic Open Source Edition was used for visual analysis of the docking results.

### Transmission Electron Microscope and Confocal Imaging

The mitochondrial ultrastructure was observed using a transmission electron microscope (Transmission Electron Microscope HT7700; Hitachi) after extracting mitochondria according to the above method. Images of cellular staining were captured using an inverted confocal laser scanning microscope (LSM 710, Carl Zeiss).

### Lentivirus construction and transfection

For Lentivirus construction, oligonucleotides with the following targeting MGAT1 were used for the cloning of small hairpin RNA–encoding(shRNA-encoding) sequences into pHBLV-U6-MCS-CMV-ZsGreen-PGK-Puro vector (purchased from HANBIO). The pHBLV-U6-shRNA-CMV-ZsGreen-PGK-Puro and PSPAX2 and PMD2G were cotransfected into HEK293 cells by using LipoFiterTM transfection reagent (HANBIO) to generate the lentivirus. The virus supernatant was collected at 48 hours and 72 hours, respectively. After collection, it was filtered with a 0.45 μm filter and centrifuged in a 40 mL ultracentrifuge tube at 4°C and 72,000*g* for 120 minutes.

Human TregNs were obtained by magnetic bead sorting according to the above method. Cells were cultivated in the presence of IL-2 (300 U/mL) for 24 hours and then transfected with viral supernatant supplemented with 4 mg/mL polybrene, followed by centrifugation for 1 hour at 1,455*g*. Afterward, cells were cultured at 37°C with 5% CO_2_ for an additional 48–72 hours.

### Substrate radioactive activity detection

C^13^- sodium lactate (Sigma-Aldrich) and C^14^- sodium lactate (PerkinElmer, IncNEC599) were added into the medium for coculture. Cells were collected at different time points and organelles were extracted according to the reagent instructions. Cell (organelle) samples were added to the measuring bottle and then 10 mL scintillation solution was added to mix evenly. After standing for 3 hours, the radioactivity was measured with a liquid scintillation counter.

### Radioactive self development

The extracted mitochondrial proteins were electrophoresed on SDS-PAGE gel. After the electrophoresis, the gel and phosphorus screen were exposed in a –80°C refrigerator for 2 weeks. After the exposure, Typhoon (GE Healthcare) was used to scan the phosphorus screen.

### Double-luciferase reporter assay

HEK293 cells were transfected with MGAT1-promoter Luc (Hanbio Biotechnology Co., Ltd.; 400 ng/well), β-gal expression vector (200 ng/ well), and XBP1s expression vectors (Hanbio Biotechnology Co., Ltd.; 200 ng/well) as indicated using lipo3000 (Sigma-Aldrich). After 24 hours, the medium was replenished and cells were cultured for an additional 24 hours. Luciferase activity was determined with a luciferase reporter assay system (Promega). β-gal activity, as the control for transfection efficiency, was measured by a kit (Mairybio Biological).

### Xeno-GvHD model generation

NOD/SCID mice lacking T cells, B cells, and NK cells were obtained from GemPharmatech Co., Ltd. After 12-hour fasting, mice received a single dose of 200 cGy of γ irradiation, followed by same-day injection of human PBMCs with or without Tregs at a dose of each 10 million cells of each type per mouse. A cohort received saline instead of human PBMCs and served as an additional control group. All experiments were performed according to the guidelines of the Institutional Animal Committee of Nanjing Medical University.

### Statistics

Statistical analysis was performed using GraphPad Prism 10.1(GraphPad Software). Significant differences were assessed by 2-tailed Student’s *t* test, 1-way ANOVA, or 2-way ANOVA. Data are presented as the mean ± SEM. All bar graphs include means with error bars to show the distribution of the data. A *P* value of 0.05 or less was considered statistically significant.

### Study approval

The PB leukopheresis product was collected by the Memorial Blood Centers and deidentified before being purchased to conduct these studies, thereby meeting approval criteria for exemption by the University of Minnesota Institutional Review Board.

Also, the ethics committee of the First Affiliated Hospital of Nanjing Medical University proved the protocol including obtaining the PBMCs from normal volunteers recruited by the First Affiliated Hospital of Nanjing Medical University (2021-SRFA-346).

### Data availability

Raw data of sequencing data were deposited in iProX (https://www.iprox.cn/page/home.html) under accession no. IPX0009884000. The values for all data points in the graphs are reported in the Supporting Data Values file.

## Author contributions

LL designed the experiments, evaluated the data, and wrote the manuscript. JZ performed the experiments, evaluated the data, and wrote the manuscript. J Gu performed the experiments and interpreted the data. QQ performed the experiments and analyzed the results. YZ provided proprietary material and reviewed the manuscript. TH, XL, ZL, and QS reviewed and discussed the data. Y Liang, LQ, XX, and QC discussed study design and interpretation of data and edited the manuscript. ZX, Y Li, J Gao, YP, YW, and ROC reviewed and discussed the data. LL, BRB, and KLH designed the overall concept, analyzed the data, and wrote the manuscript. The order of the cofirst authors was assigned based on their efforts and contributions to the study.

## Supplementary Material

Supplemental data

Unedited blot and gel images

Supplemental video 1

Supplemental video 2

Supporting data values

## Figures and Tables

**Table 1:**
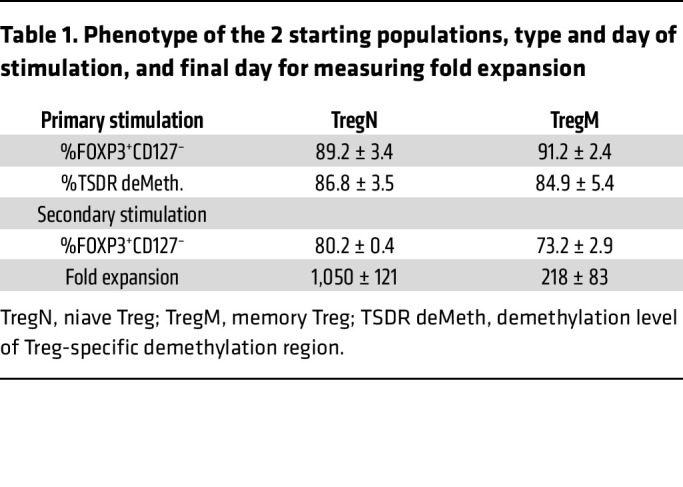
Phenotype of the 2 starting populations, type and day of stimulation, and final day for measuring fold expansion

**Table 2:**
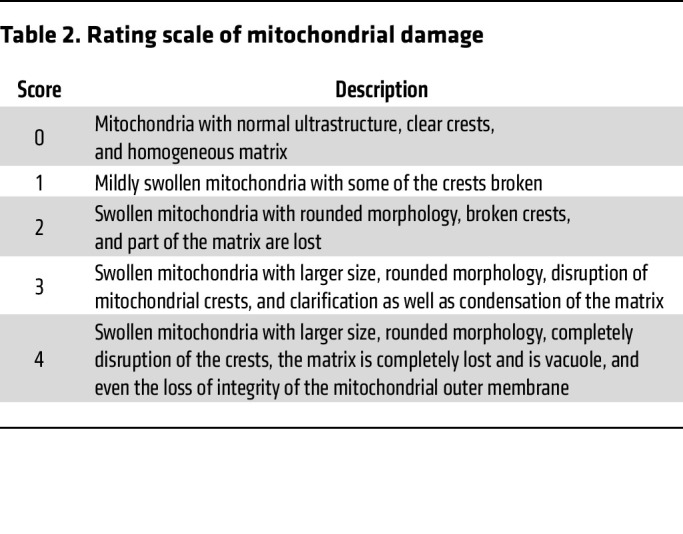
Rating scale of mitochondrial damage

## Version 1 09/12/2024

In-Press Preview

## Version 2 10/15/2024

Electronic publication
