# Supplementary material for: Lactate supports Treg function and immune balance via MGAT1 effects on N-glycosylation in the mitochondria
Source: J Clin Invest. 2024 Sep 12;134(20):e175897. doi: 10.1172/JCI175897 (PMC11473165; doi:10.1172/JCI175897)
Supplement: Supplemental data [file jci-134-175897-s167.pdf]

Supplemental Figure

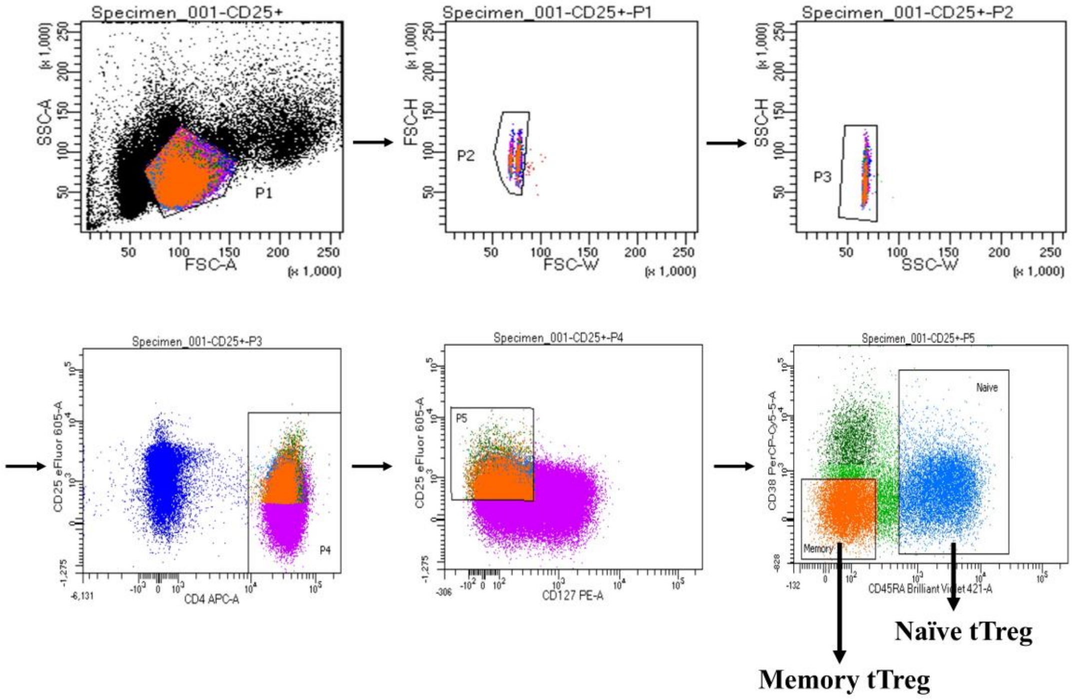

SFigure 1. Sorting process of TregN and TregM

TregN (CD4$^+$25$^+$127$^-$45RA$^+$) and TregM (CD4$^+$25$^+$127$^-$38$^-$45RA$^-$) were sorted from human PBMCs by FACS before exploring the effect of high-concentration lactate on TregN and TregM *ex vivo* expansion.

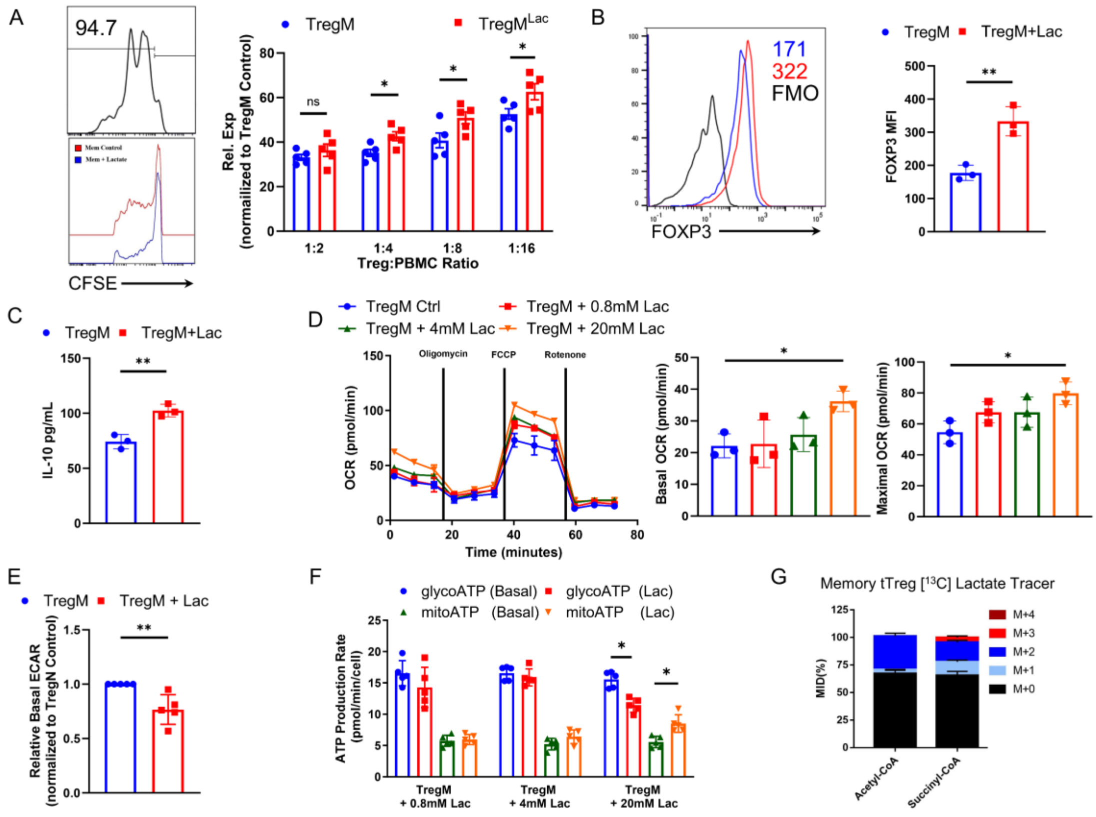

SFigure 2. The effect of lactate on phenotype and metabolism of TregM

(A) Inhibitory effect of Treg cells on PBMC proliferation as assessed by flow cytometry (TregM were stimulated with anti-CD3/CD28 mAb-coated beads, and cocultured with CFSE-labeled PBMC cultured in X-VIVO 15 media with and without 20 mM lactate for 48 hours). n=5.

(B) MFI of FOXP3 in TregM with and without lactate treatment for 48 hours assessed by flow cytometry. n=3

(C) Secretion of IL-10 in TregM with and without lactate treatment for 48 hours by flow cytometry. n=3

(D) OCR of TregM was measured by Seahorse assay (TregM were stimulated with anti-CD3/CD28 mAb-coated beads and treated with different doses of lactate for 24 hours). n=3

(E) Basal ECAR of TregM with and without lactate treatment. n=5

(F) Mitochondrial-derived and glycolysis-derived ATP production following different doses of lactate treatment in TregM by ATP rate assay. n=5

(G) Tricarboxylic acid derivative analysis showed that TregM integrated lactate into the Krebs cycle (TregM were harvested after one-day stimulation with anti-CD3/CD28 mAb-coated beads and treated with Sodium L-lactate-^{13}C for an additional one hour). Data are represented as mean ± SEM. Two-tailed Student's t test (B, C, E, and F) or one-way ANOVA (D). *$p < 0.05$, **$p < 0.01$, ns, not significant.

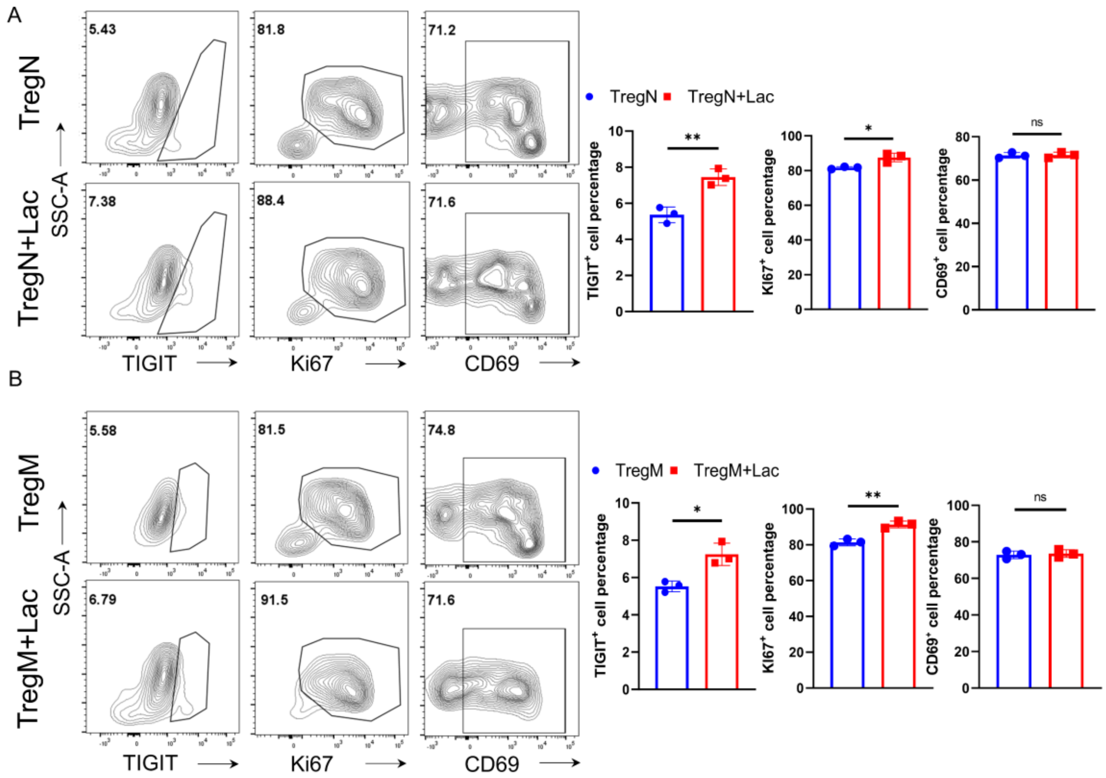

SFigure 3. Treg-associated marker expression after lactate treatment.

(A) TIGIT, Ki67, and CD69 Expression in TregN with and without lactate treatment for 48 hours. n=3.

(B) TIGIT, Ki67, and CD69 expression in TregM with and without lactate treatment for 48 hours. n=3.

Data are represented as mean ± SEM. Two-tailed Student's t test. *p < 0.05, **p < 0.01. ns, not significant.

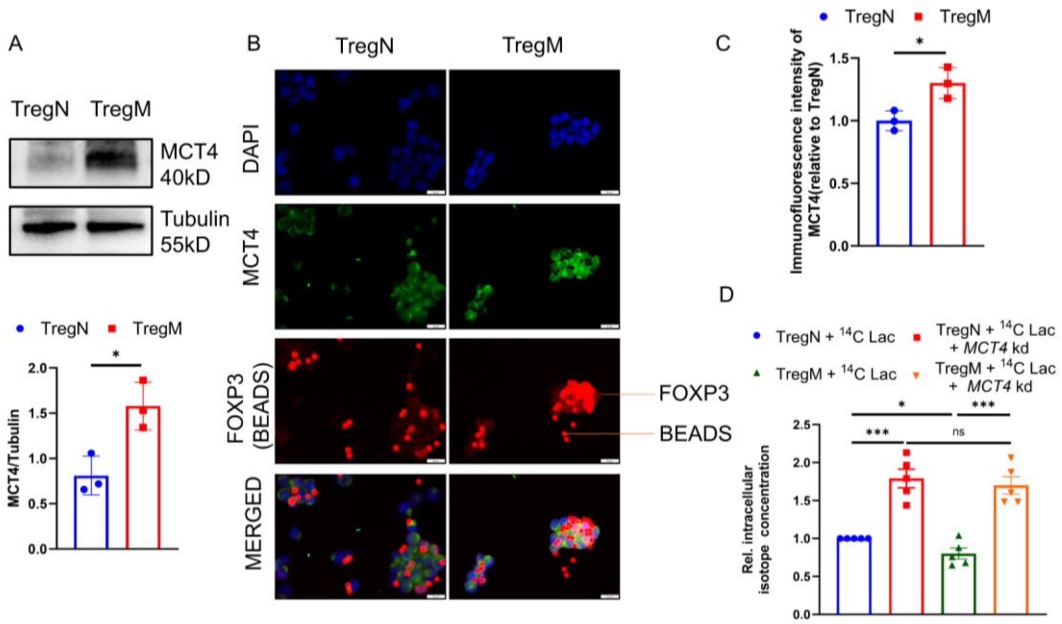

SFigure 4. Lactate concentration is higher in TregN than in TregM.

(A) Expression of MCT4 in TregN and TregM (cultured for 48 hours) by Western Blot. n=3.

(B) Expression of MCT4 in TregN and TregM (cultured for 48 hours) under laser scanning confocal microscopy (Red fluorescence could be observed for FOXP3 and anti-CD3/CD28 mAb-coated beads at the same time, in which small, deeply dyed spheres were beads).

(C) Representative histogram of the immunofluorescence intensity of MCT4 in (B). n=3.

(D) 5μl C^{14}- sodium lactate was used to measure intracellular isotope concentration of TregN, TregM, TregN with *MCT4* knockdown, and TregM with *MCT4* knockdown. n=5.

Data are represented as mean ± SEM. Two-tailed Student's t test. $^{*}p < 0.05$, $^{***}p < 0.001$, ns, not significant.

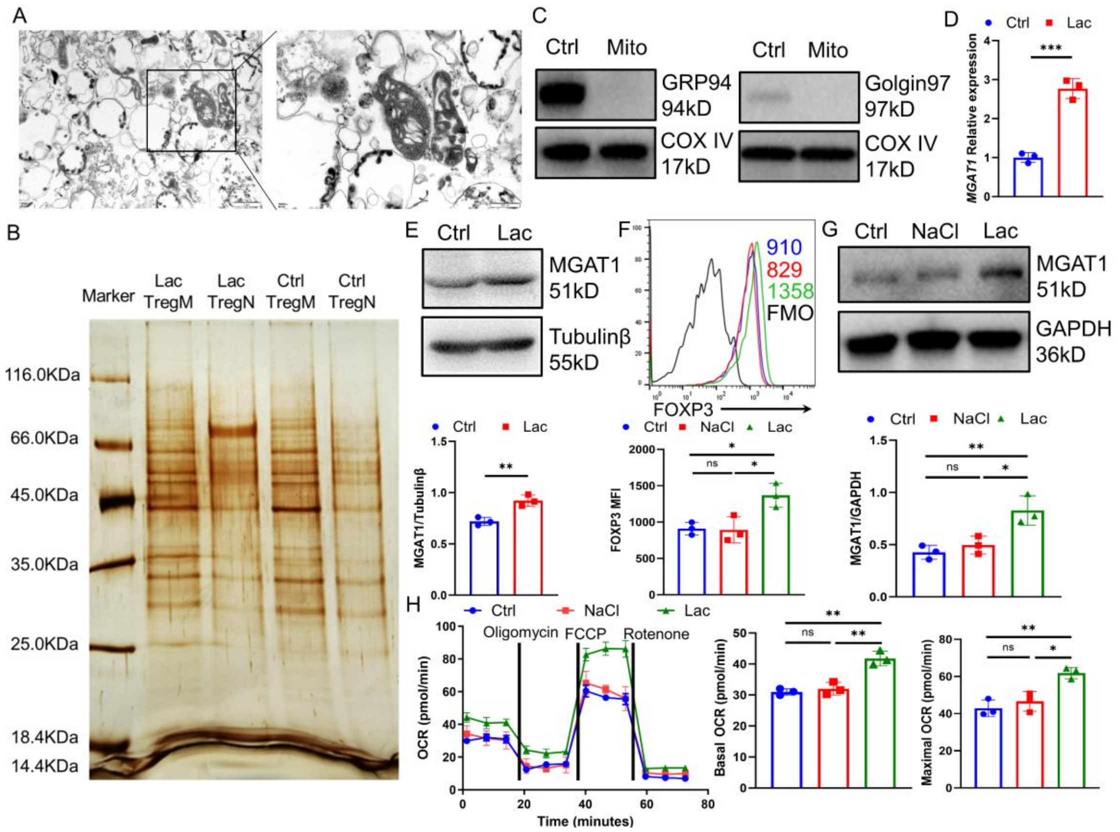

SFigure 5. Isolated mitochondrial protein quality and the effects of NaCl on TregN

(A) Morphology of mitochondria after isolation under the transmission electron microscope.

(B) Silver staining of mitochondrial proteins in TregN and TregM with or without lactate treatment by Western blot (2 μg/well).

(C) Expression of Golgin97 (Golgi apparatus) and GRP94 (endoplasmic reticulum) in the whole cell and isolated mitochondria of TregN by Western blot.

(D) Expression of *MGAT1* mRNA in TregM after 20 mM lactate treatment for 48 hours. n=3

(E) MGAT1 expression in TregM after 20 mM lactate treatment for 48 hours. n=3

(F) MFI of FOXP3 in three groups: TregN, TregN with NaCl, and TregN with lactate treatment for 48 hours by flow cytometry. n=3

(G) Expression of MGAT1 in groups in (F) for 48 hours by Western Blot. n=3

(H) OCR of groups in (F) for 24 hours by Seahorse assay. n=3

Data are represented as mean ± SEM. Two-tailed Student’s t test (D and E) or one-way ANOVA (F-H). $^{*}p < 0.05$, $^{**}p < 0.01$, $^{***}p < 0.001$.

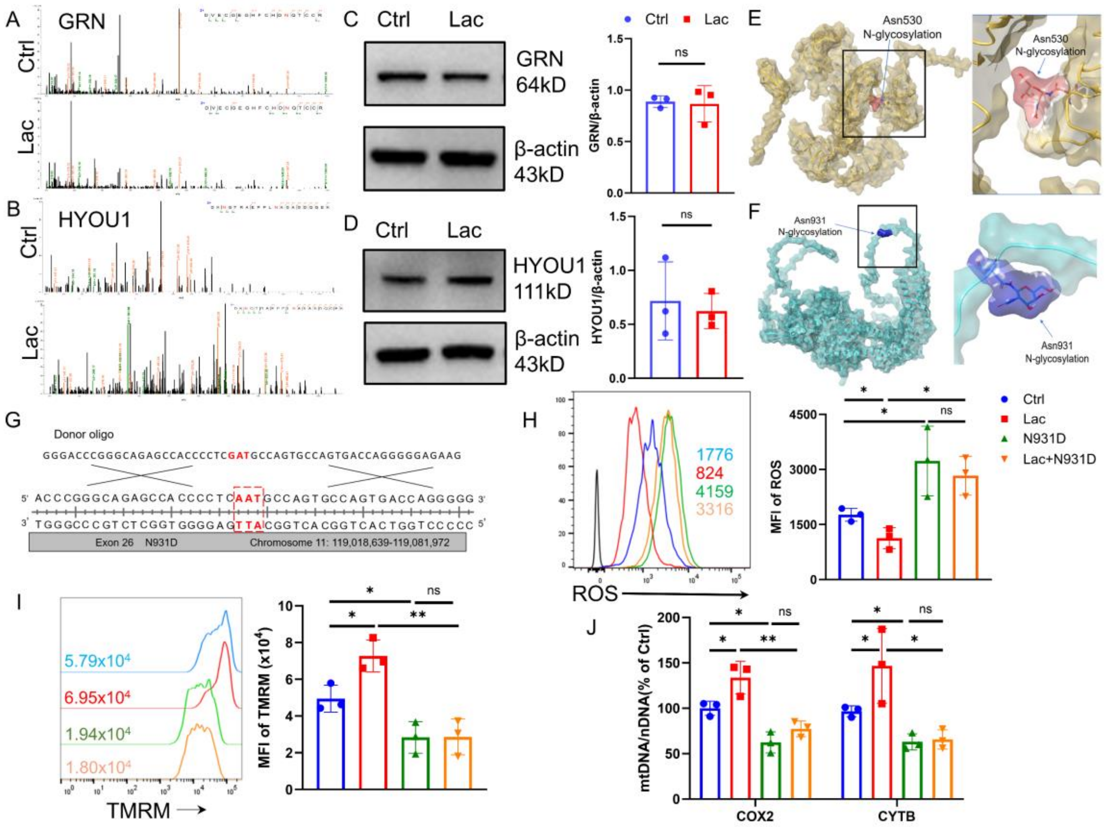

SFigure 6 Lactate treatment increases N-glycosylation of GRN and HYOU1 to promote Treg OXPHOS

(A) The second-order spectrum of GRN (N530) in the control and lactate group.

(B) Second-order mass spectrum of HYOU1 (N931) in the control and lactate groups.

(C) GRN expression in TregN with or without lactate treatment for 48 hours by Western blot. n=3.

(D) HYOU1 expression in TregN with or without lactate treatment for 48 hours by Western blot. n=3.

(E) Potential effect of N-glycosylation on GRN (N530) by predicting the structure before and after N-glycosylation modification using three-dimensional modeling.

(F) Potential effect of N-glycosylation on HYOU1 (N931) by predicting the structure before and after N-glycosylation modification using three-dimensional modeling.

(G) CRISPR-Cas9 gene editing technology was used in Jurkat to mutate the Asparagine at HYOU1 N931 to Aspartic acid in vitro.

(H) Fluorescence signal intensity of ROS in four groups after 48 hours culture: Jurkat, lactate-treated Jurkat, HYOU1 N931 mutation Jurkat, and lactate treatment with HYOU1 N931 mutation Jurkat. n=3.

(I) TMRM fluorescence signal intensity of Jurkat in (D). n=3.

(J) Mitochondrial to nuclear DNA (mtDNA: nDNA) ratio of four groups in (D). n=3.

Data are represented as mean ± SEM. Two-tailed Student's t test (C and D) or One-way ANOVA(H-J). *$p < 0.05$, **$p < 0.01$, ns, not significant.

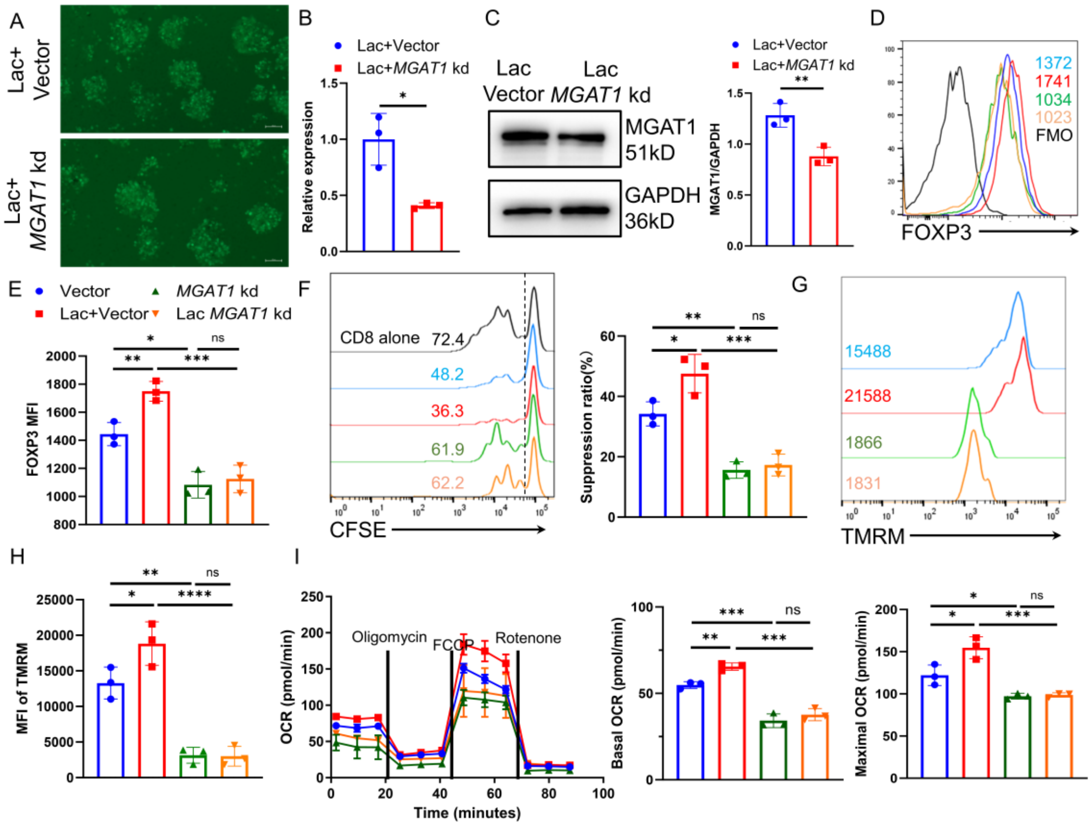

SFigure 7. Mitochondrial function of TregM declines after *MGAT1* knockdown

(A) Transfection efficiency of lentivirus in lactate with lentiviral vector and lactate with *MGAT1* knockdown group for three days was shown by microscopy images in TregM.

(B) Expression of *MGAT1* mRNA in groups in (A). n=3.

(C) Expression of MGAT1 in groups in (A) by Western blot. n=3.

(D) Representative FOXP3 MFI by flow cytometry in four groups: lentiviral vector, lactate with lentiviral vector, *MGAT1* knockdown, and lactate with *MGAT1* knockdown group for three days.

(E) Analysis of FOXP3 MFI in four groups in (D). n=3.

(F) Inhibitory function of TregM in four groups in (D) on CD8^{+} T cell proliferation (CD8: TregM = 2:1) by flow cytometry. n=3.

(G) Representative TMRM fluorescence signal intensity by flow cytometry in four groups in (D).

(H) Analysis of TMRM fluorescence signal intensity by flow cytometry in four groups in (D). n=3

(I) OCR of TregM in four groups in (D) via cell metabolism measurement (Seahorse assay).

Data are represented as mean ± SEM. Two-tailed Student’s t test (B and C) or one-way ANOVA (E-I). *$p < 0.05$, **$p < 0.01$, ***$p < 0.001$, ****$p < 0.0001$. ns, not significant.

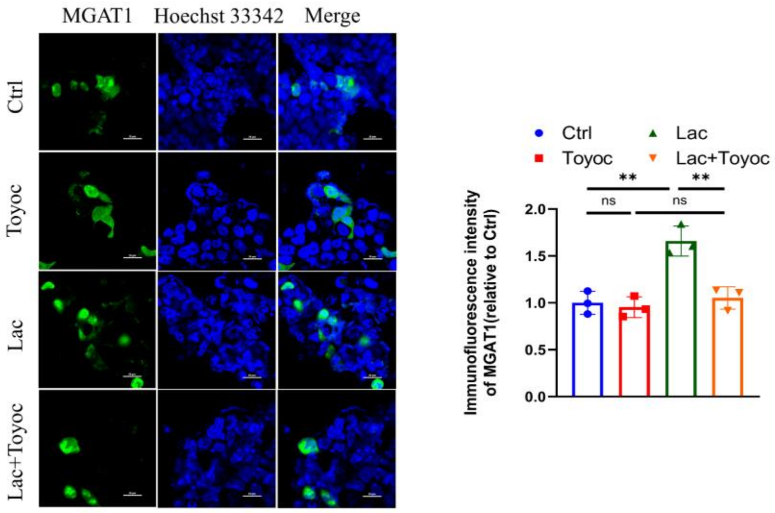

SFigure 8. Effects of toyocamycin and lactate on the immunofluorescence intensity of MGAT1 of TregN under the laser scanning confocal microscopy.

Data are represented as mean ± SEM. One-way ANOVA. **$p < 0.01$, ns, not significant.

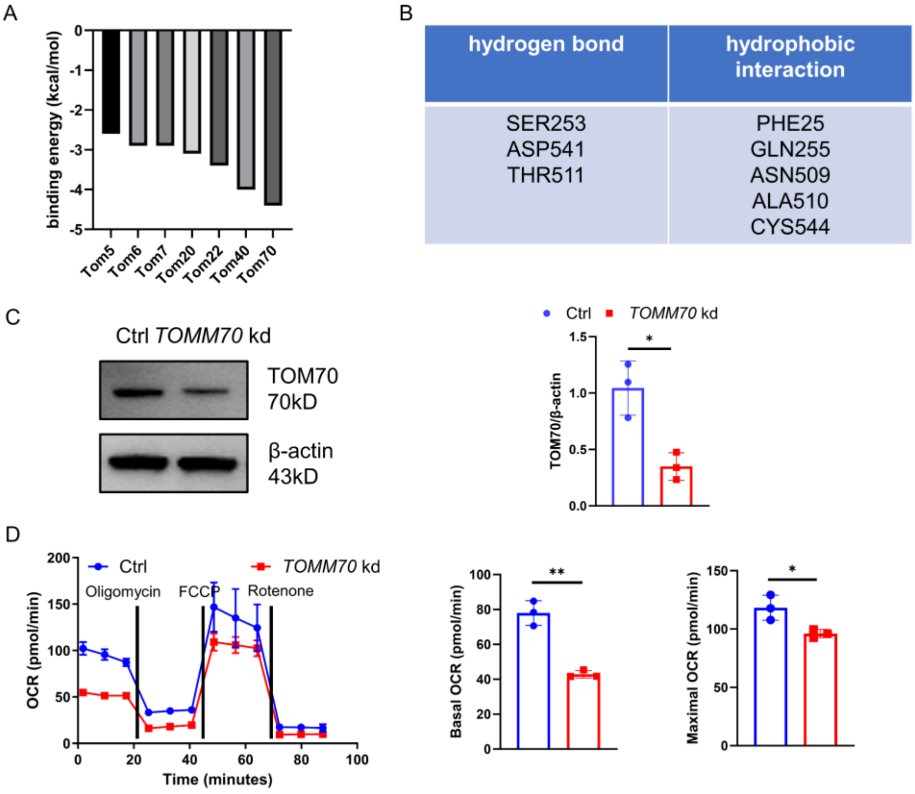

SFigure 9. TOM70 is essential for mitochondrial translocation of MGAT1 in TregN in a high lactate environment

(A) Binding affinity of lactate and TOM family proteins was performed by AutoDock Vina (SMINA) software.

(B) Potential contributing amino acids related to the complex of TOM70-lactate.

(C) Expression of TOM70 after *TOMM70* knockdown by Western blot. n=3.

(D) OCR of TregN after *TOMM70* knockdown by Seahorse assay. n=3.

Data are represented as mean ± SEM. Two-tailed Student's t test. *p < 0.05, **p < 0.01.

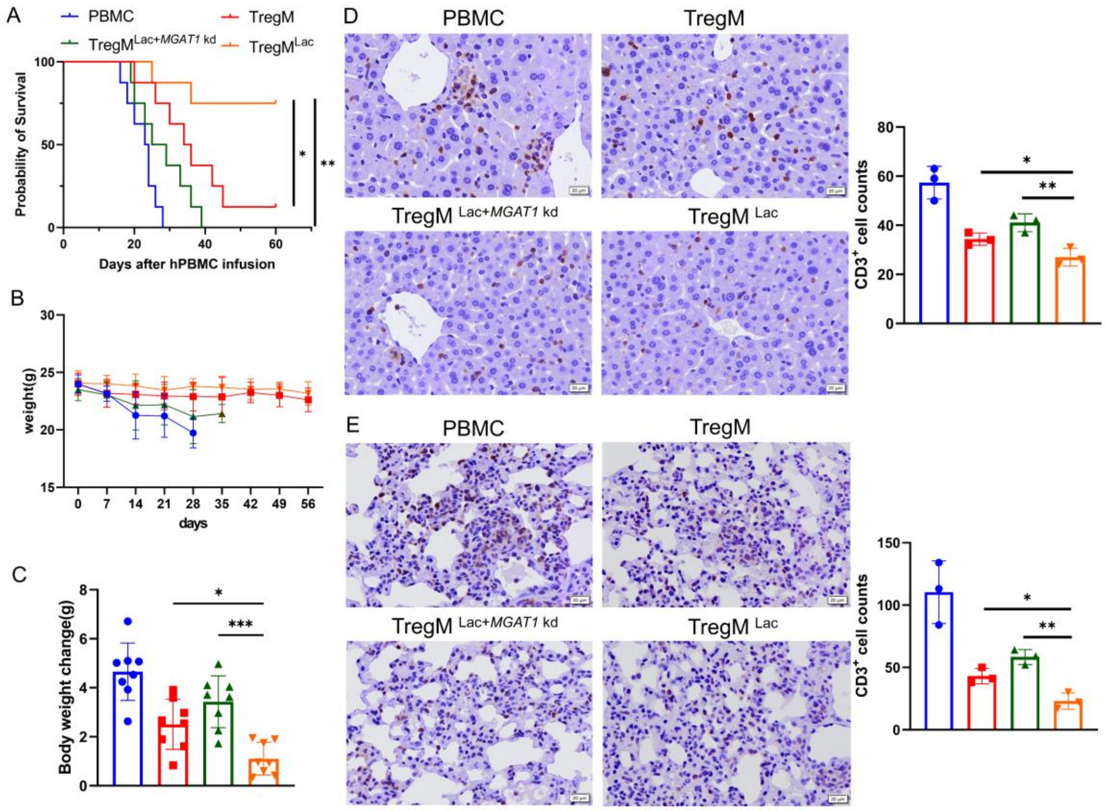

SFigure 10. *MGAT1* knockdown attenuates the therapeutic effect of TregM on GvHD

(A) The survival of GvHD mice within 60 days injected with PBMC, PBMC with untreated TregM, PBMC with lactate-treated TregM, and PBMC with lactate-treated *MGAT1* knockdown TregM via tail vein (10 million cells). n=8.

(B) Weight change of GvHD mice within 60 days in (A). n=8.

(C) Representative histogram of Weight change of GvHD mice within 60 days in (A). n=8.

(D) $CD3^+$ T cell infiltration in the liver of GvHD mice in (A) by immunohistochemistry. n=3.

(E) $CD3^+$ T cell infiltration in the lung of GvHD mice in (A) by immunohistochemistry. n=3.

Data are represented as mean ± SEM. Statistical analysis was performed using one-way ANOVA (C-E) or two-way ANOVA (A). *$p < 0.05$, **$p < 0.01$, ***$p < 0.001$.
