# Supplementary material for: Lactate supports Treg function and immune balance via MGAT1 effects on N-glycosylation in the mitochondria
Source: J Clin Invest. 2024 Sep 12;134(20):e175897. doi: 10.1172/JCI175897 (PMC11473165; doi:10.1172/JCI175897)

Figure2F

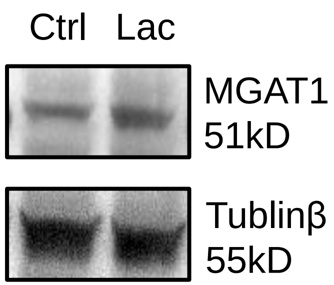

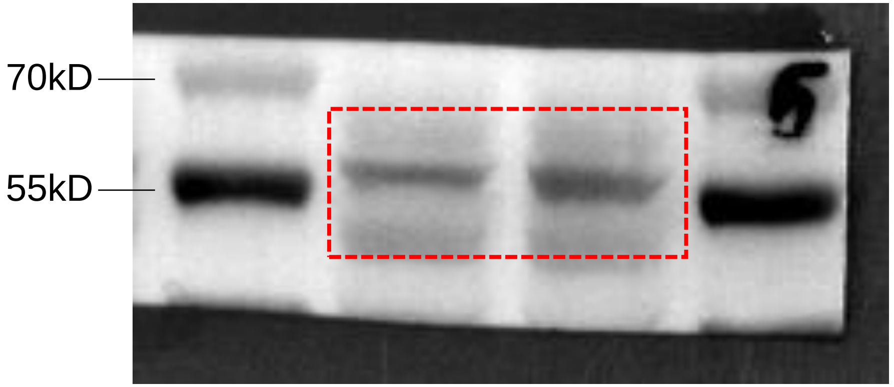

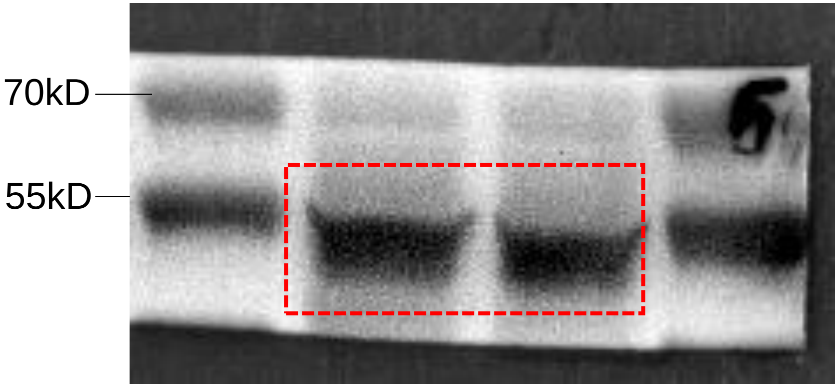

SFigure4A

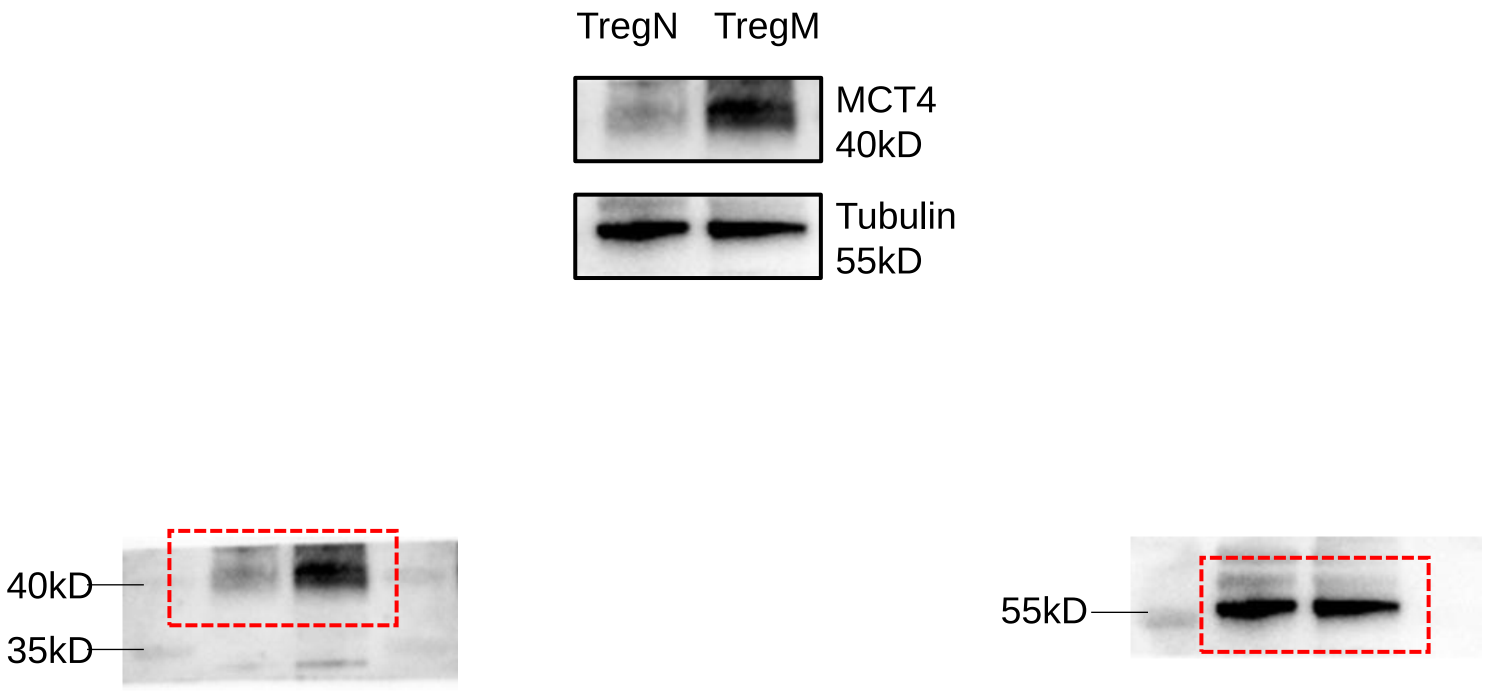

SFigure5C

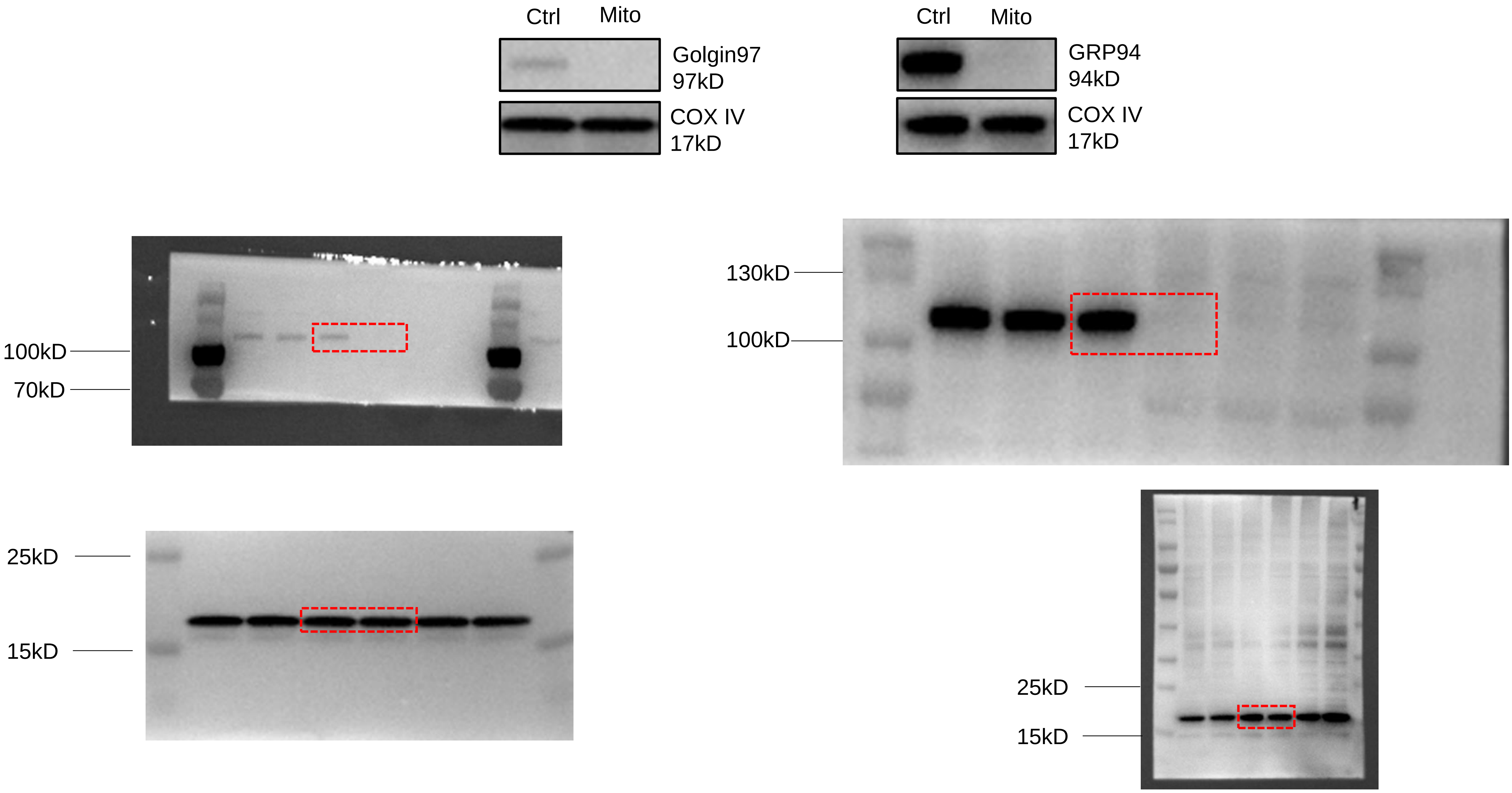

Ctrl
Mito
Golgin97
97kD
COX IV
17kD
Ctrl
Mito
GRP94
94kD
COX IV
17kD
100kD
70kD
130kD
100kD
25kD
15kD
25kD
15kD

SFigure5E

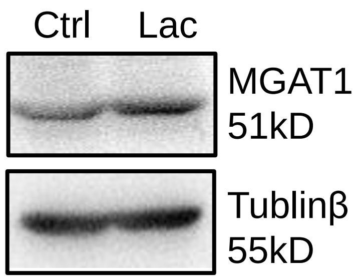

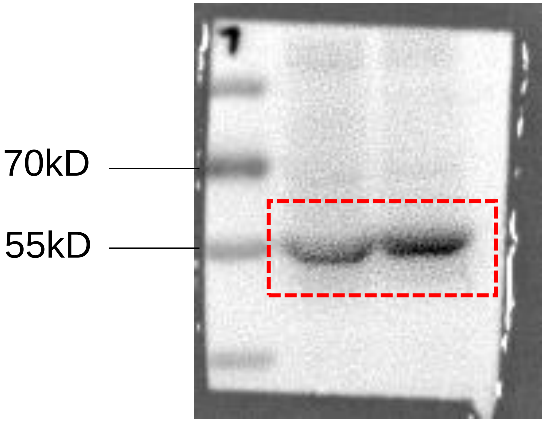

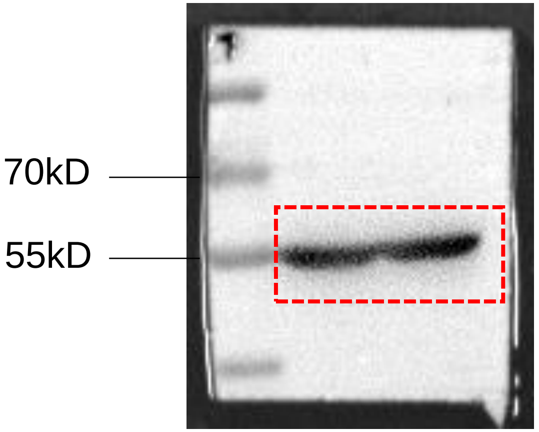

SFigure5G

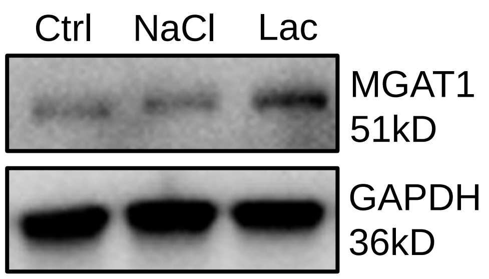

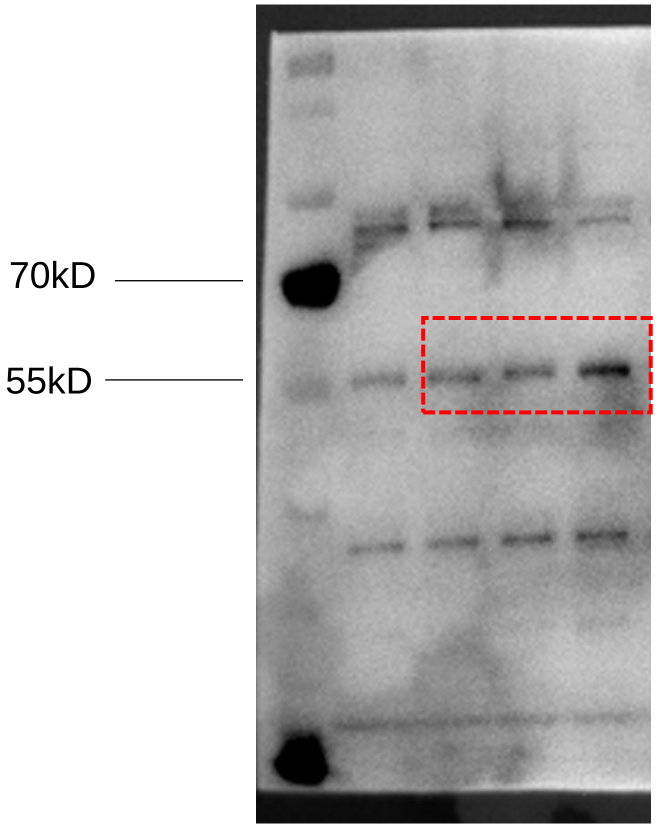

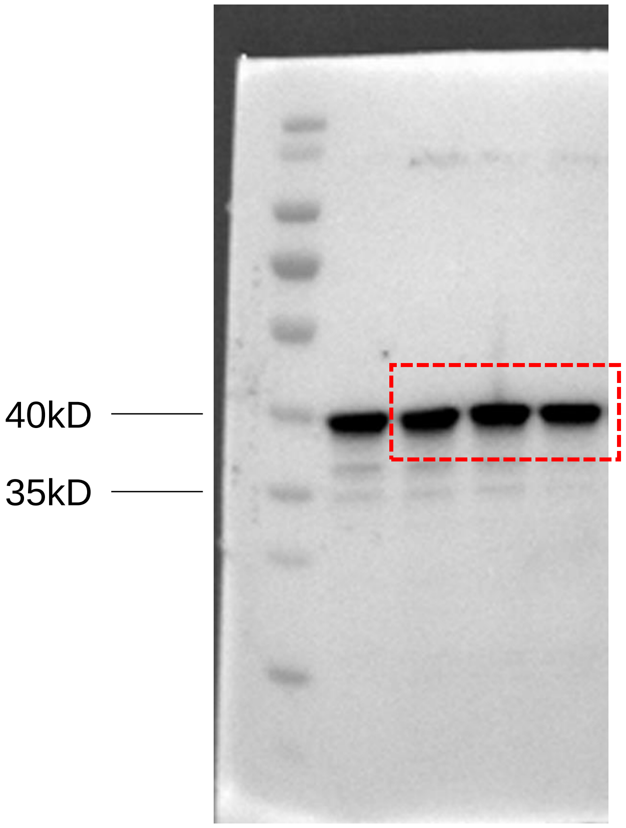

SFigure6C-D

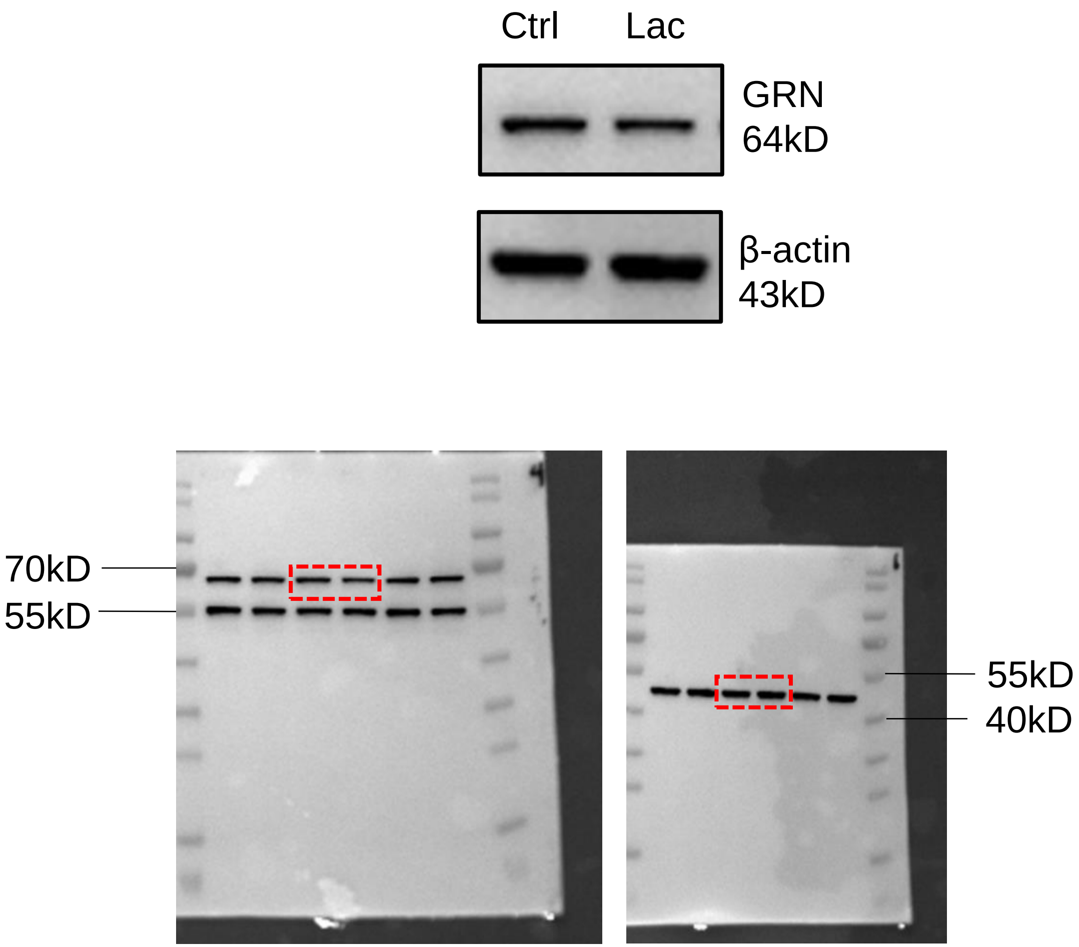

Ctrl
Lac
GRN
64kD
β-actin
43kD
70kD
55kD
55kD
40kD

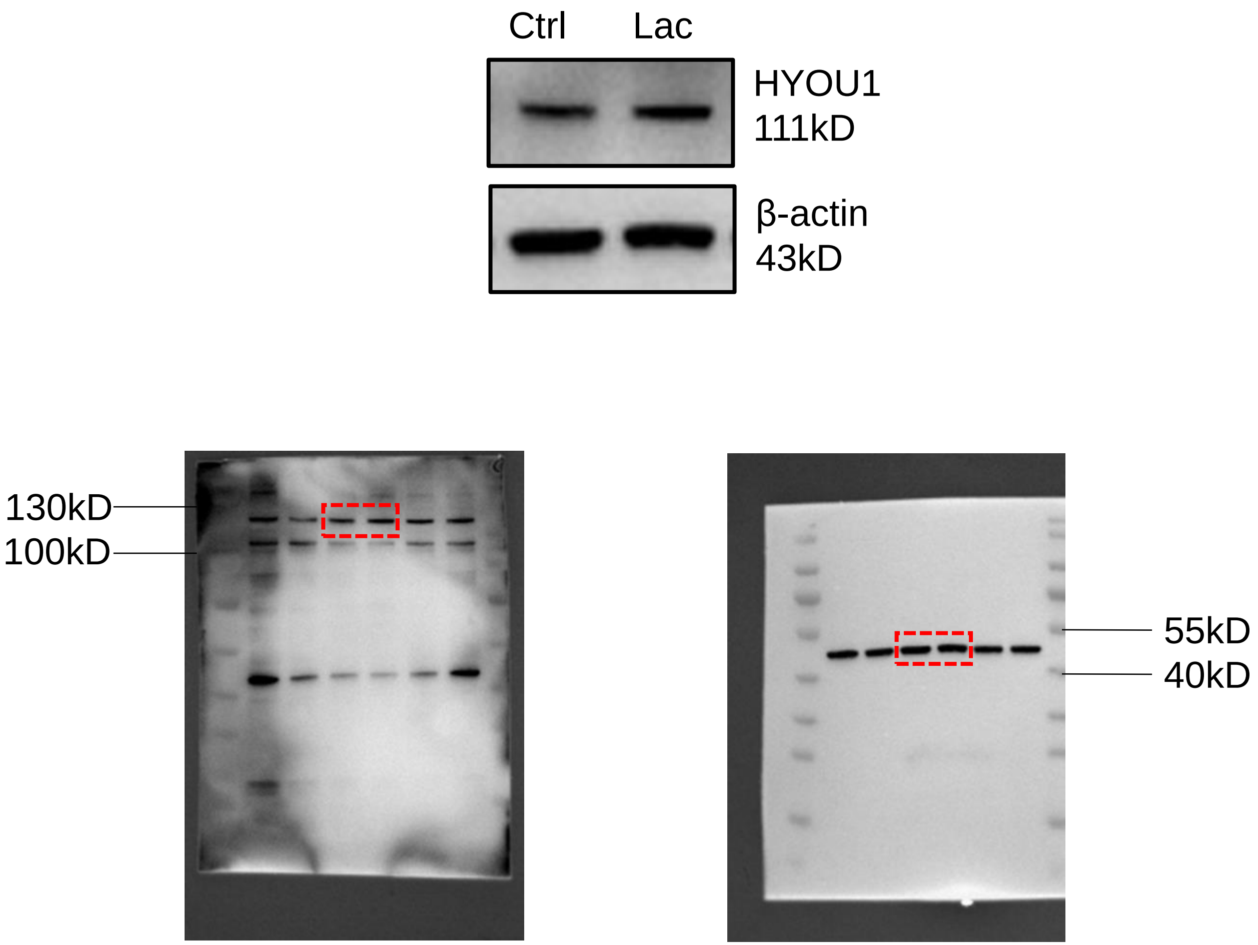

Ctrl
Lac
HYOU1
111kD
β-actin
43kD
130kD
100kD
55kD
40kD

Figure4C and SFigure7C

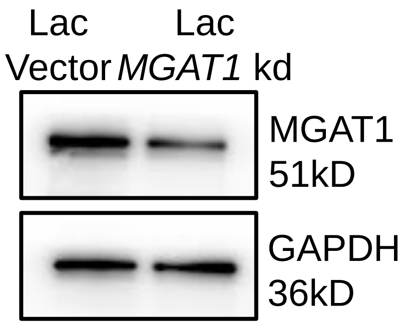

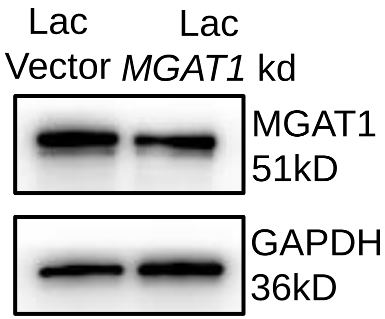

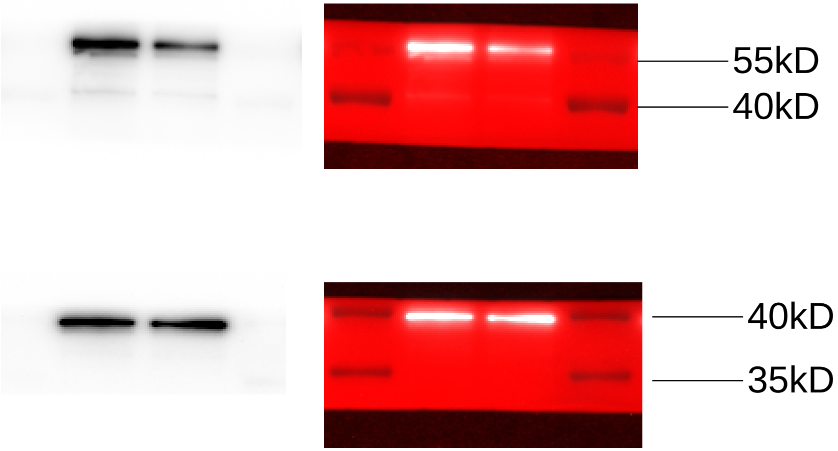

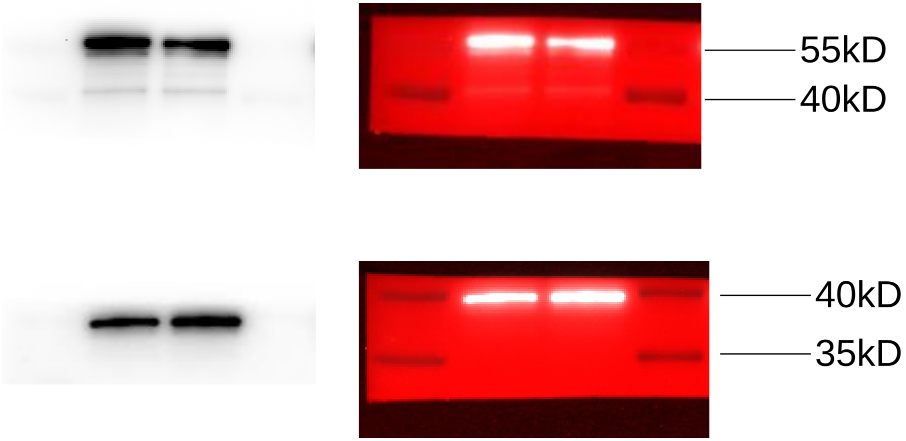

Figure5F

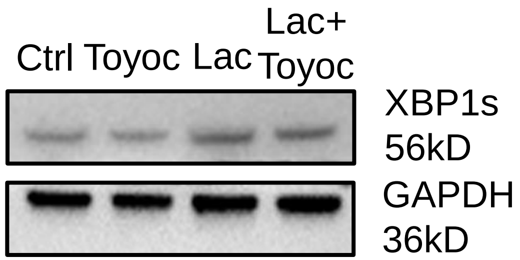

Lac+
Ctrl Toyoc Lac Toyoc
XBP1s
56kD
GAPDH
36kD

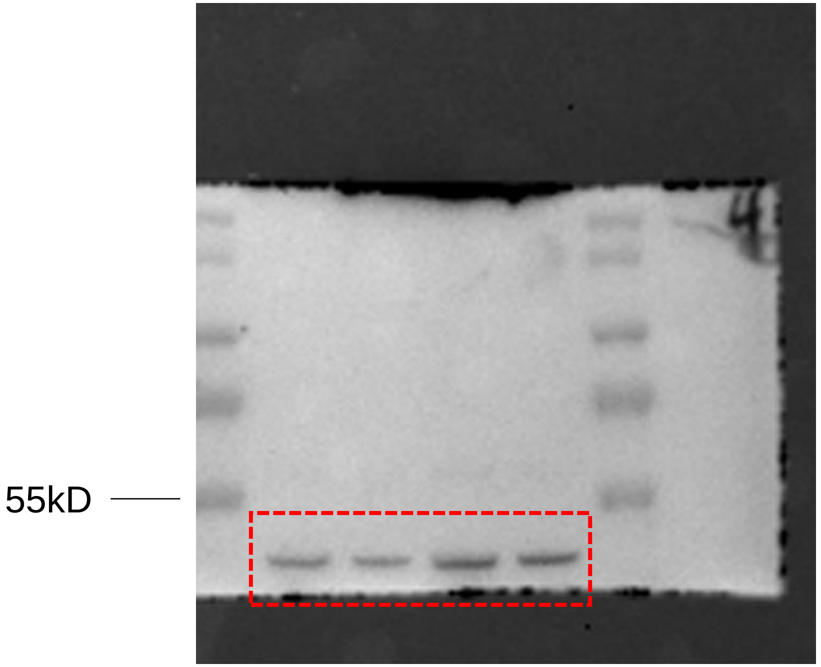

55kD

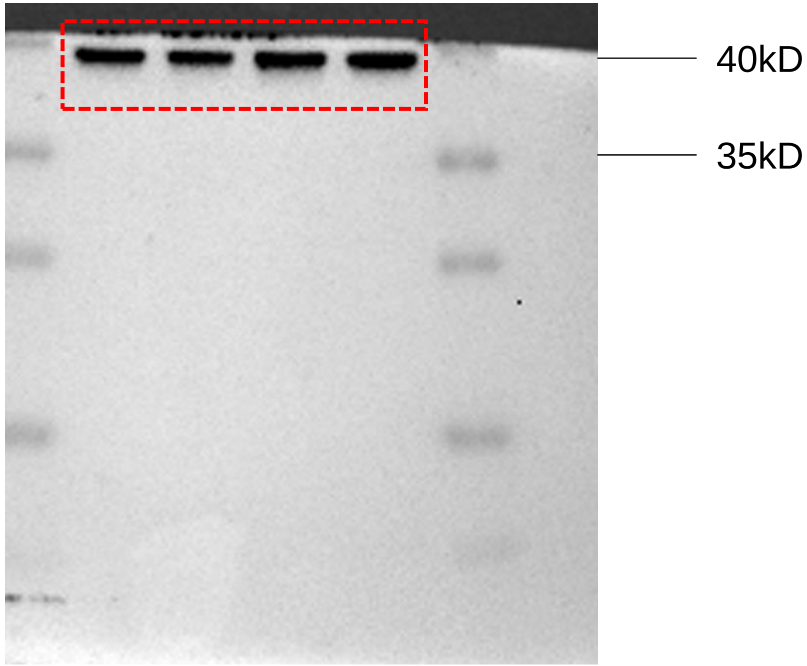

40kD
35kD

Figure6C

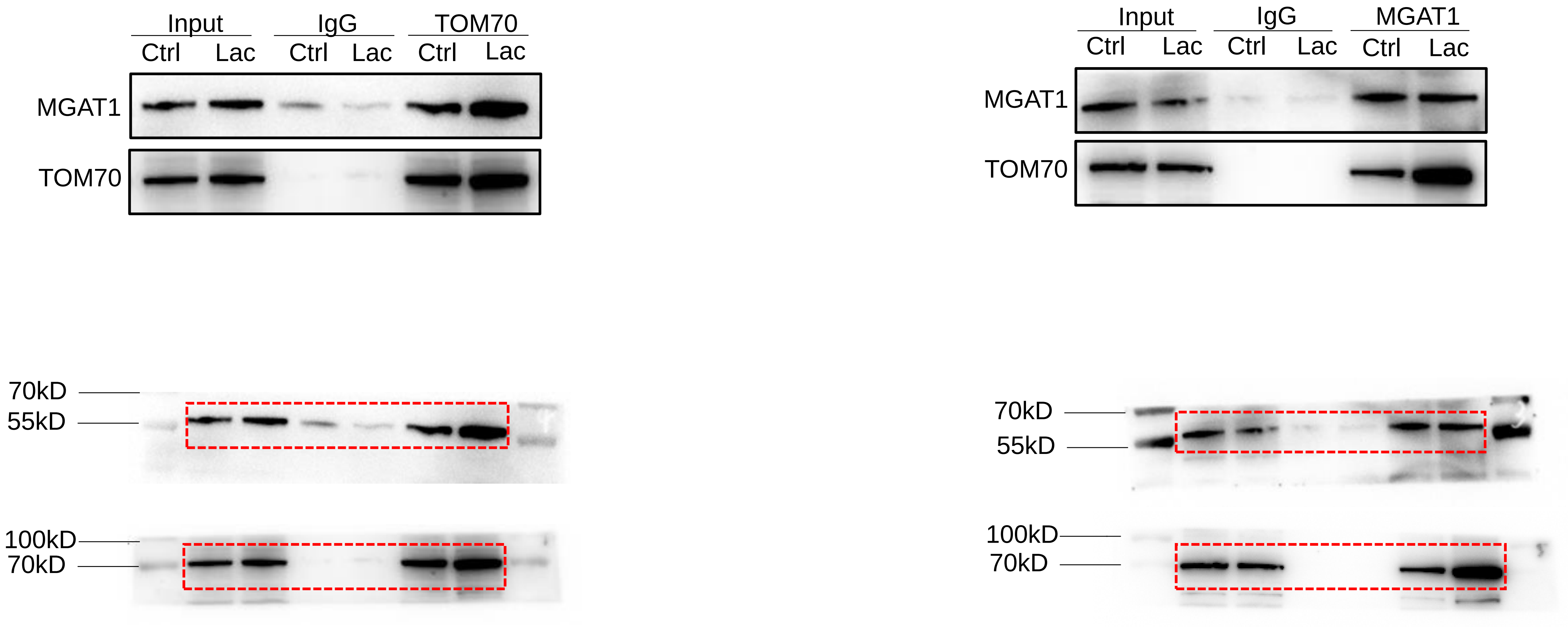

Input
IgG
TOM70
Ctrl
Lac
Ctrl
Lac
Ctrl
Lac
MGAT1
TOM70
Input
IgG
MGAT1
Ctrl
Lac
Ctrl
Lac
Ctrl
Lac
MGAT1
TOM70
70kD
55kD
100kD
70kD
70kD
55kD
100kD
70kD

Figure6D

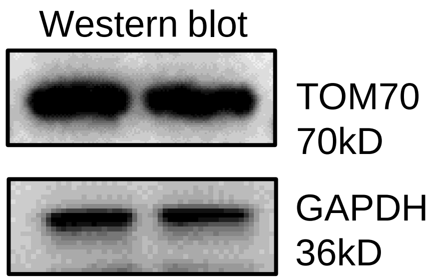

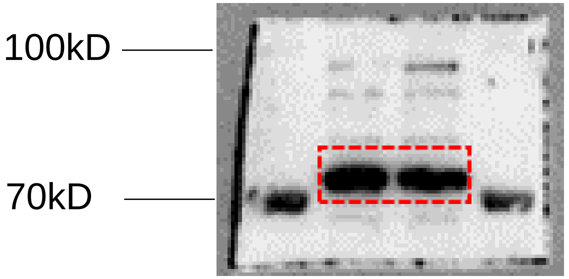

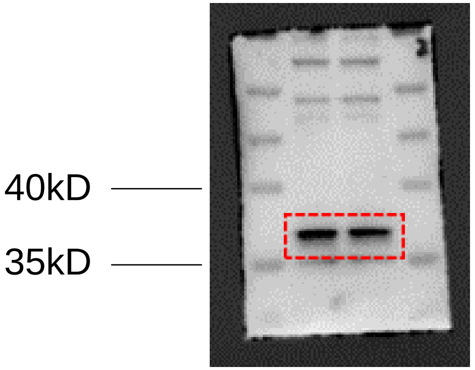

Figure6F

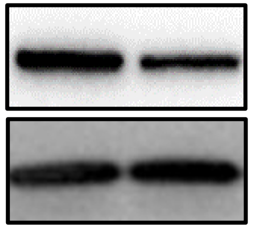
Vector
TOMM70 kd
MGAT1 51kD
COX IV 17kD

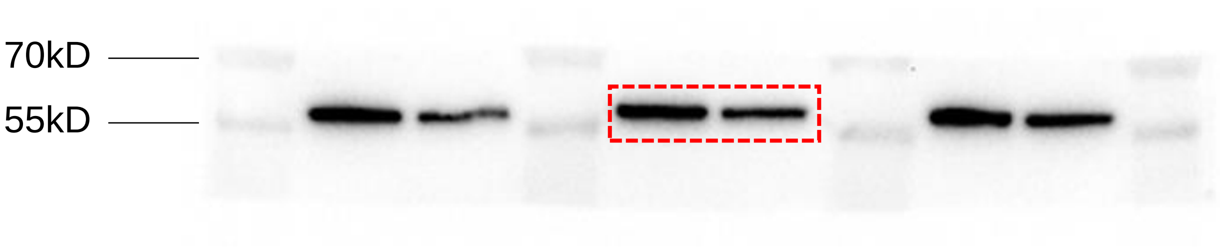
Vector
TOMM70 kd
Vector
TOMM70 kd
Vector
TOMM70 kd
70kD
55kD

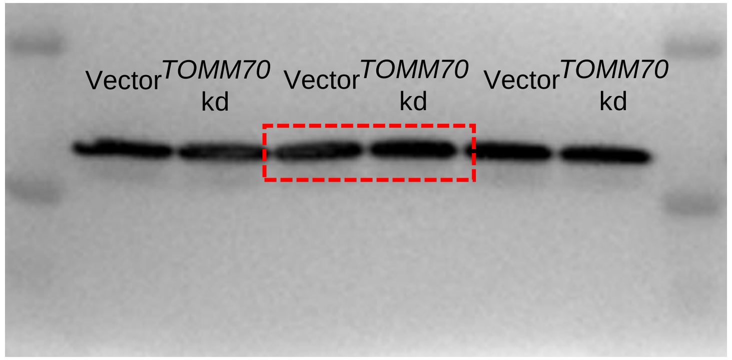
25kD
15kD
Vector
TOMM70 kd
Vector
TOMM70 kd
Vector
TOMM70 kd

SFigure9C

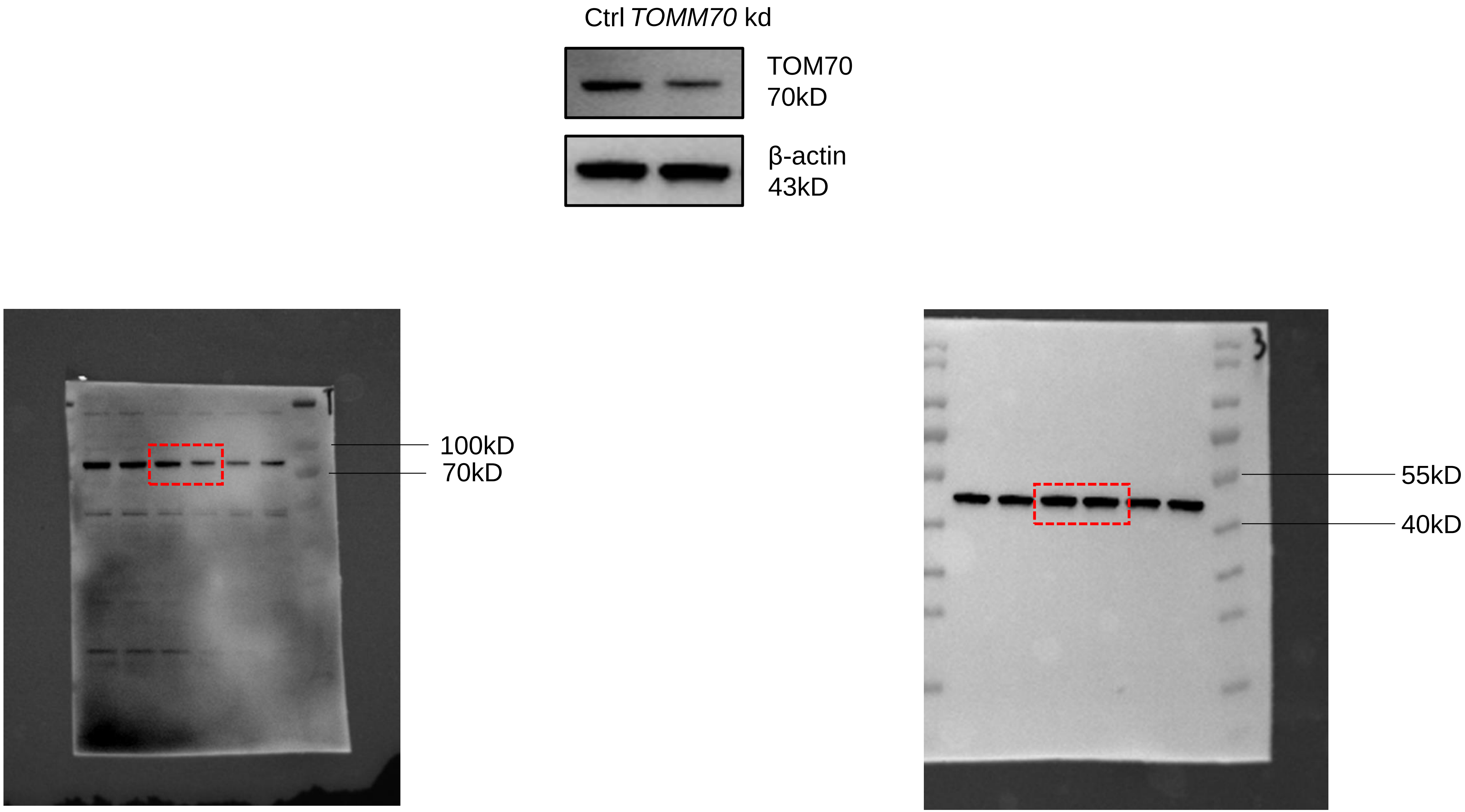

Supplement: Unedited blot and gel images [file jci-134-175897-s168.pdf]
